# Paris Climate Agreement passes the cost-benefit test

**DOI:** 10.1038/s41467-019-13961-1

**Published:** 2020-01-27

**Authors:** Nicole Glanemann, Sven N. Willner, Anders Levermann

**Affiliations:** 1 Potsdam Institute for Climate Impact Research, 14473 Potsdam, Germany; 2 WHU—Otto Beisheim School of Management, 56179 Vallendar, Germany; 3 Institute of Physics, Potsdam University, 14476 Potsdam, Germany; 4 Columbia University, New York, NY 10027 USA

**Keywords:** Environmental economics, Economics

## Abstract

The Paris Climate Agreement aims to keep temperature rise well below 2 °C. This implies mitigation costs as well as avoided climate damages. Here we show that independent of the normative assumptions of inequality aversion and time preferences, the agreement constitutes the economically optimal policy pathway for the century. To this end we consistently incorporate a damage-cost curve reproducing the observed relation between temperature and economic growth into the integrated assessment model DICE. We thus provide an inter-temporally optimizing cost-benefit analysis of this century’s climate problem. We account for uncertainties regarding the damage curve, climate sensitivity, socioeconomic future, and mitigation costs. The resulting optimal temperature is robust as can be understood from the generic temperature-dependence of the mitigation costs and the level of damages inferred from the observed temperature-growth relationship. Our results show that the politically motivated Paris Climate Agreement also represents the economically favourable pathway, if carried out properly.

## Abstract (web summary)

Relative economic benefits of achieving temperature targets have not properly accounted for damages at higher temperatures. Here the authors integrate dynamic cost-benefit analysis with a damage-cost curve and show that the Paris Climate Agreement constitutes the economically optimal policy pathway for the future.

## Introduction

The temperature targets as agreed upon in the Paris Climate Agreement^1^ result from a long and complex political process^2^. However, it is not clear whether the associated emission reduction efforts are economically favourable^2,3^. Although econometric analyses^4–8^ suggest large damages at higher temperatures, these have not yet been employed to derive the relative economic benefits of achieving these temperature targets^2,3^. In particular, estimates^6,8^ of observed temperature-induced losses in gross domestic product have not been accounted for in computations of the economically optimal policy pathways. Here we provide a macroeconomic assessment of these targets by accounting for recent estimates of warming-induced economic growth impacts, which are given by Burke et al.^6,8^ (BHM, hereafter). BHM have advanced prior knowledge^4^ on the relationship between temperature und economic growth by finding a universal non-linear relationship. Warming is shown to lead to a shift along the growth curve and to reduce growth beyond a certain temperature threshold.

So far, the BHM estimates have been shown to correspond to rather high social cost of carbon^9^, indicating that emission reduction should be stringent. However, the implications for optimal policy have only been investigated along predetermined scenarios of warming and economic growth^6,8–10^. Although such estimates are not without criticism^9,11^, it is a natural and necessary next scientific step to compare them to the costs of mitigating climate change (mitigation costs, hereafter) using an integrated assessment model (IAM). IAMs account for the diverse dynamic interactions between the economy and the climate^12,13^.

**Fig. 1:**
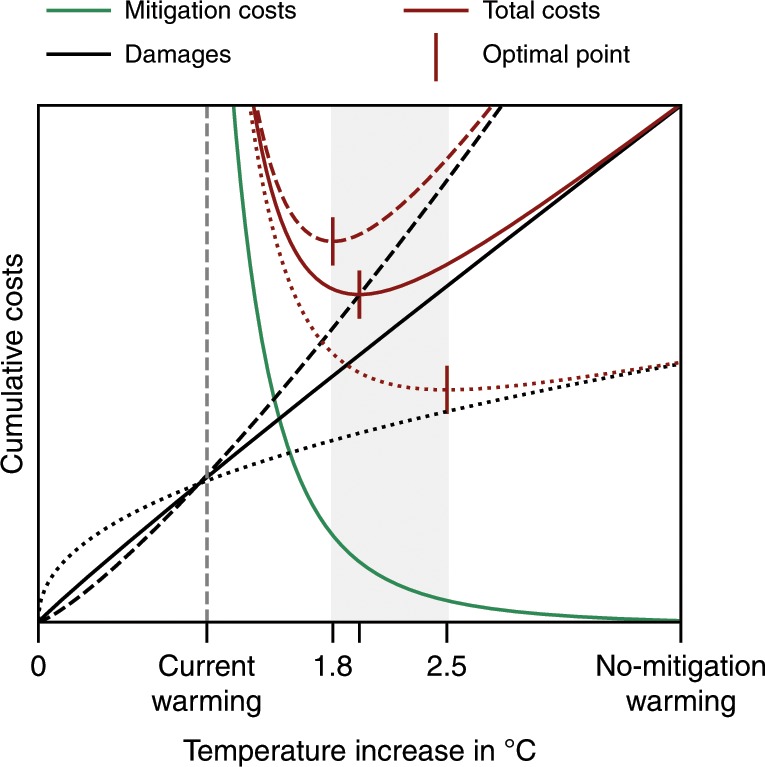
Illustration of universality of the cost-benefit climate analysis. Cumulative mitigation costs (green curve) and climate damages (black curve) as a function of Earthʼs warming level give the total climate costs (red curve). Mitigation costs diverge for present-day warming and converge to zero for unmitigated warming. The damages are zero for zero warming and increase with temperature. The characteristic steepness of the mitigation curve implies that beyond a certain damage level the economically optimal temperature (which minimizes the total costs) becomes insensitive to a further increase in damages. For example, increasing (black dashed) or decreasing (black dotted) the damage level by half of the initial damage level does not change the economically optimal warming level significantly (grey area).

This comparison provides the end-of-century warming that is associated with the lowest total costs of damages and mitigation as employed in the IAM used (Fig. 1). Cost-benefit optimal warming is thus determined by the shape of the two cost curves. The mitigation-cost curves are characterized by two universal properties. First, they diverge at the present-day warming, in particular if negative-emission technologies are not available. Second, the mitigation costs decrease to zero for a warming scenario without any mitigation efforts. The damage-cost curve, on the other hand, is known to be zero without warming and to increase with rising temperatures. The level to which the damages rise without mitigation is subject to investigation. However, due to the divergence of the mitigation costs the economically optimal temperature becomes less sensitive to the exact level of damages once these have reached a certain level (Fig. 1). Here, we examine whether the damages that follow from extrapolating the observed relation of economic growth and temperature^6,8^ are beyond this level.

To this end we incorporate the BHM estimates into one of the most prominent IAMs^14–16^, DICE-2013^16^. With its simplicity, DICE allows assessing cost-benefit optimality in a scientifically highly transparent and controlled way. According to its original version, which has also been employed to advise US climate policy^17–19^ achieving the 2 °C target would cause mitigation costs significantly larger than the consequent avoided damages^16,20,21^. This result is largely due to a damage function that does not incorporate recent estimations of economic impacts^13,22,23^. Here, we update this function according to the BHM estimates^8^. As DICE searches for the economic growth path that maximises global welfare, the growth estimates cannot be implemented directly. As a solution to this problem we develop a novel procedure that preserves the growth model feature. In that, we iteratively adjust the damage function to reproduce the estimated temperature-induced growth relation in DICE-2013. For consistency with the BHM estimates, we design a scenario that emulates a future world in which key conditions are similar as in the past, i.e. the absence of climate policy.

We use this updated damage function to derive the cost-benefit optimal climate policy that begins with the year 2020. In this economically optimal scenario, mitigation is actively pursued to maximize global welfare. We continue holding the assumption of DICE-2013 that significant negative-emission technologies are not available in this century. We contrast the optimal policy with the business-as-usual (BAU) scenario, in which climate policy is absent. We find that under these conditions the 2 °C target as set by the Paris Climate Agreement gives the cost-benefit optimal pathway till the end of this century. We observe that this finding is largely robust to diverse uncertainties. Our results thus advocate for rapid and decisive implementation of the Paris Climate Agreement.

## Results

### Cost-benefit optimal temperature

In our analyses, we account for uncertainty in the future temperature development by considering three alternative equilibrium climate sensitivity (ECS) values. In addition, we subject our results to extensive robustness tests. We examine the effects of uncertainty in the BHM estimates concerning the parameter values and the model specification. For this we adopt the bootstrapping approach from the original empirical study^8^ and use the resulting 1000 samples to derive a corresponding ensemble of damage functions. We also conduct a sensitivity analyses regarding social preferences for consumption changes^24^, alternative socioeconomic futures^25^, and mitigation costs.

**Fig. 2:**
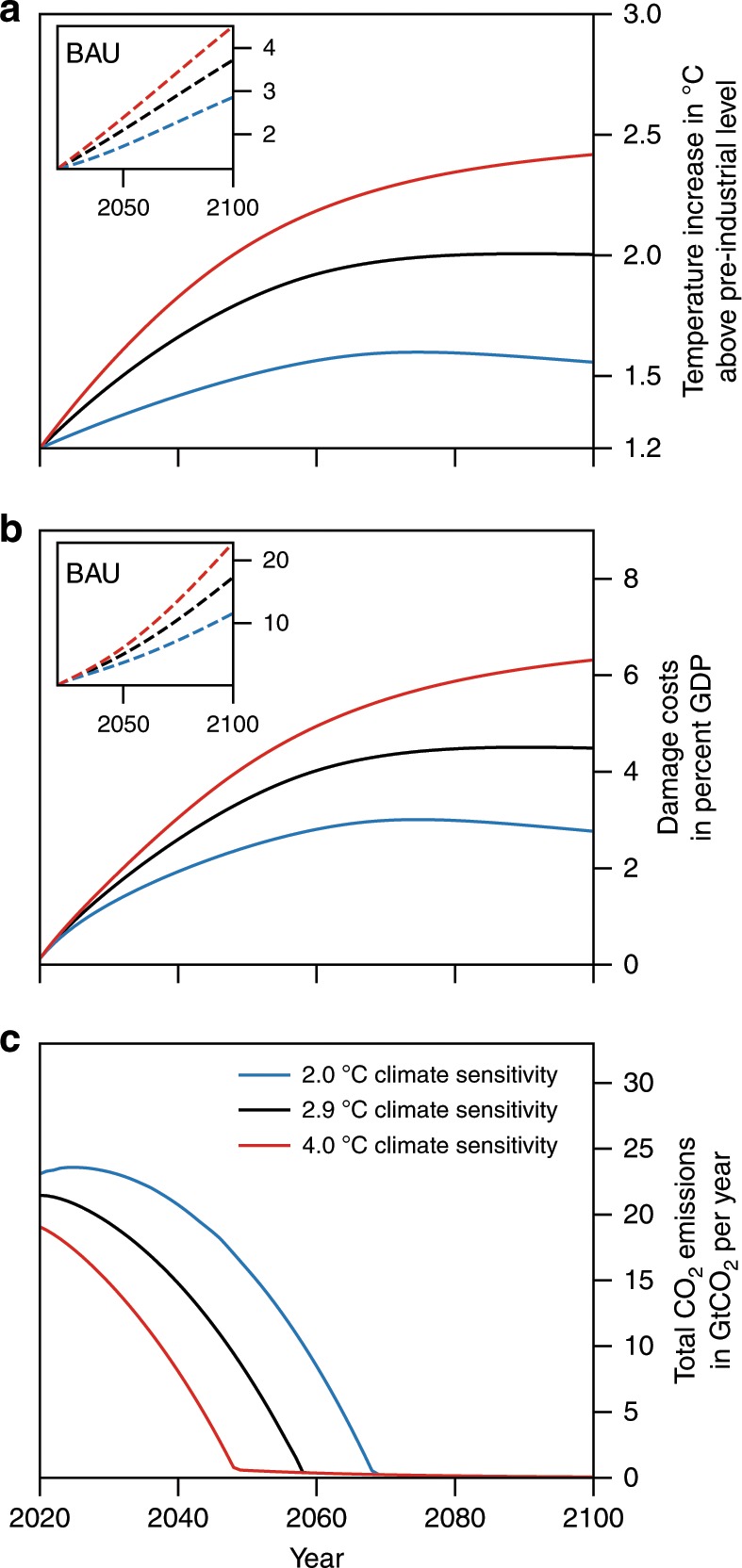
Temperature increase, damage costs, and carbon emissions under cost-benefit optimal policy for three different climate sensitivities. The black curves are associated with the original calibration of the climate sensitivity of 2.9°C; the blue curves with a 2°C climate sensitivity and the red curve with a 4°C climate sensitivity. The inset figures allow comparing the economically optimal temperature development and damage costs with their corresponding values in the BAU scenario.

We find that the 2 °C target represents the cost-benefit optimal temperature for the base calibration (Fig. 2a). This calibration involves the best estimate^8^ of the temperature–economic growth relation in the past and the original ECS value in DICE-2013 of 2.9 °C, which is at the centre of estimates for several decades^26,27^. Higher ECS values shift the level of target warming for which the mitigation-cost curve diverges to infinity to higher values (Fig. 1), i.e. they incur substantially higher mitigation costs. For ECS of 4 °C, for instance, the 2 °C target becomes too costly. Yet, with an optimal target warming of 2.4 °C the deviation from this target is not large. For smaller ECS values, e.g. of 2 °C, limiting warming further to well below 2 °C is economically optimal. Regardless of the exact ECS, the optimal mitigation efforts promise a significant damage reduction compared to the BAU scenario (~14% for ECS of 4 °C, ~10% for ECS of 2.9 °C, and ~8% for ECS of 2 °C). These efforts are, as also claimed by the Paris Agreement, ambitious (Article 3)^1^ and involve very stringent measures from the outset (Fig. 2c).

### Uncertainty in damage function

**Fig. 3:**
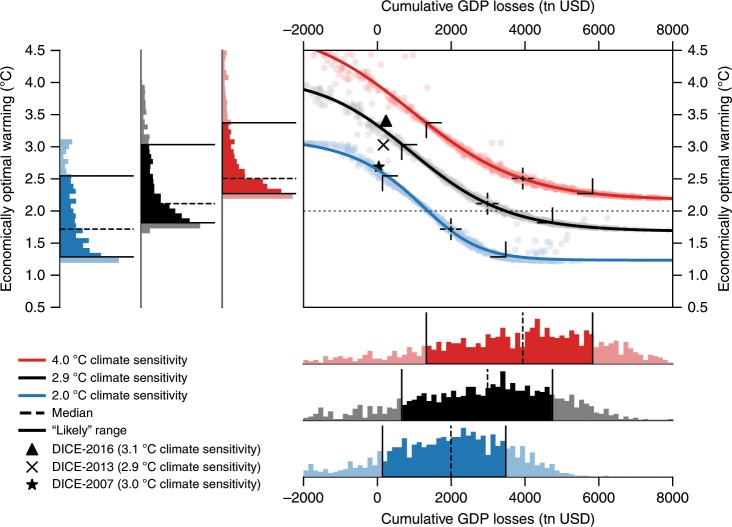
Relation between the cumulative GDP losses until 2100 (in 2005 $US) in the absence of climate policy and the economically optimal warming until the end of the century, given uncertainty in the estimates of the historical impact and uncertainty in the climate sensitivity value. Scattered points give the uncertainty ensemble in the historical relation between temperature increase and economic growth for three different climate sensitivities; red points for 4 °C climate sensitivity, black points for the original climate sensitivity calibration in the DICE-2013R model, and blue points for 2 °C climate sensitivity. Each point depicts the DICE-2013 model output for a damage function calibrated according to one of the 1000 bootstraps of the historical regression. Curves in the main plot represent the best fit for the relation between cumulative damage costs and optimal warming. The histograms below and on the left give the frequency of the model results as well as the medians and likely ranges for each of the three climate sensitivities. The likely rage of optimal end-of-century warming is approximately located between 2.3 °C and 3.4 °C with a median of 2.5 °C for the climate sensitivity of 4 °C, between 1.8 °C and 3 °C with a median of 2.1 °C for a climate sensitivity of 2.9 °C and between 1.3 °C and 2.5 °C with a median of 1.7 °C for a climate sensitivity of 2 °C. The results of the original DICE versions are located outside the likely ranges as shown by the black brackets.

**Fig. 4:**
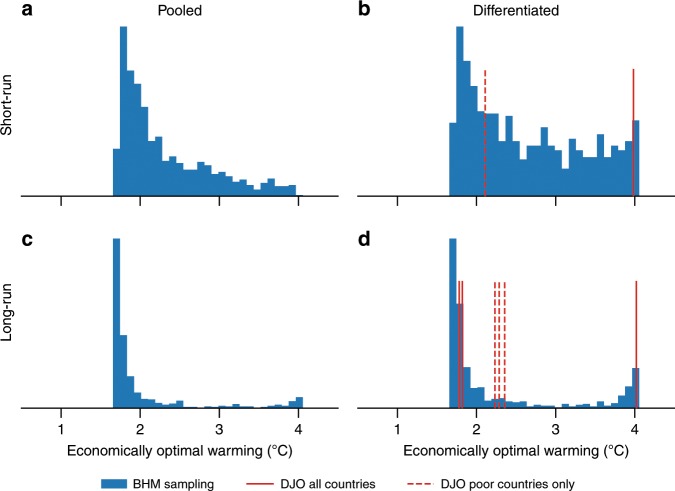
Ensembles including the uncertainty in the estimates of the historical impacts according to BHM (blue bars) and some samples according to Dell et al.^4^ (DJO, red lines). Specification of the estimates without (short-run (**a**, **b**)) and with (long-run (**c**, **d**)) the assumption that the influence of warming on economic growth is lagged and/or without (pooled (**a**, **c**)) and with (differentiated (**b**, **d**)) differentiating between impacts on poor and on rich countries. Each specification for BHM samples from 1000 bootstraps of the historical regression; samples for DJO include specifications with no lag (**b**) as well as 1-lag, 5-lag, and 10-lag specifications (**d**).

To examine the effects of uncertainty in the impact estimates, we use the cumulative GDP losses until 2100 (in 2005 $US) in the BAU scenario as a measure for the impact severity and pair them with the economically optimal end-of-century temperature (Fig. 3). The uncertainty in the damage costs, according to the empirical study^6,8^, is substantial with respect to the magnitude and sign of the warming impact and also implies large differences in our results. Nonetheless, the ensemble median of the optimal temperatures is only marginally higher than 2 °C for ECS of 2.9 °C, and well below 2 °C for ECS of 2 °C. This result is robust to alternative specifications of the bootstrapping approach^8^ (Supplementary Figs. 1 and 2) and to most alternative model specifications of BHM and the alternative econometric estimates by Dell et al.^4^ (Fig. 4 and Supplementary Figs. 3–6). Hence, the goal to limit warming to 2 °C or less is cost-benefit optimal for a wide set of damage functions. By contrast, the results of the original DICE versions^16,21^ deviate significantly from the computed likely range (Fig. 3).

### Uncertainty regarding preferences

**Fig. 5:**
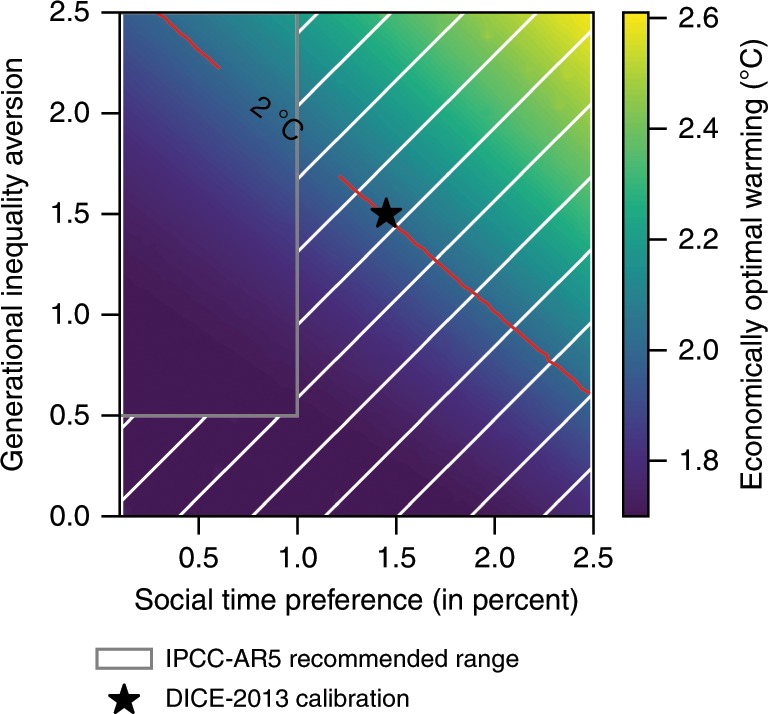
Sensitivity of the economically optimal temperature in 2100 to alternative initial rates of social time preference and generational inequality aversion. These simulations are based on the benchmark impact estimate as in Fig. 2 with an equilibrium climate sensitivity (ECS) of 2.9 °C. The unhatched box indicates the range of values recommended by the IPCC-AR5 report^29^. The black star depicts the DICE-2013^16^ calibration. The red line marks the 2 °C isoquant.

We also test the sensitivity to two important preference parameters (Fig. 5). First, the ‘initial rate of social time preference’ (IRSTP) which reflects the preference for consumption at different points in time, with a higher value giving more emphasis to present rather than to future consumption; and second, the ‘elasticity of marginal utility of consumption’ (EMUC) which describes the preferences for more consumption, irrespective of its timing, and is interpreted as generational inequality aversion^21^. As these parameters crucially affect decisions of optimal mitigation and investment^28^, the implied growth effects are critical for our results. Taking the prescriptive viewpoint of calibrating IRSTP and EMUC^24^, we account for wide value ranges, including the base calibration in DICE-2013 and the suggestions by the IPCC-AR5^29^. The latter proposes near-zero IRSTP values, which we interpret as values smaller than 1%. With the exception of a few unusual parameter values, this wide range of options leads to optimal warming of around 2 °C or lower (Fig. 5).

### Cost-benefit optimal temperature under SSP scenarios

**Fig. 6:**
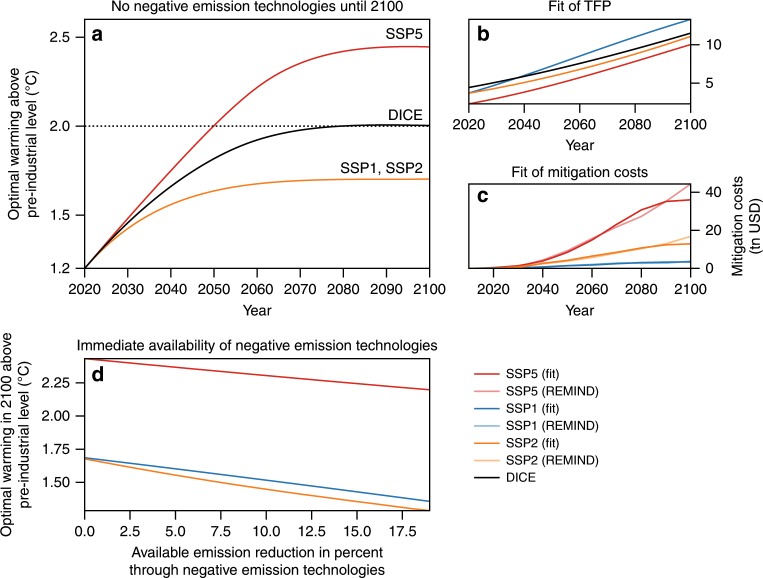
Economically optimal warming for SSP1, SSP2, SSP5 and DICE. **a** The economically optimal temperature pathway for different socioeconomic conditions under the assumption that negative-emission technologies are not used within this century. **b**, **c** Recalibrated parameters in DICE to match the results of the REMIND model for the three SSPs. **b** shows the results for the total factor productivity (TFP) and **c** for the costs of mitigation. **d** Economically optimal warming in 2100 if negative-emission technologies are available in this century.

**Fig. 7:**
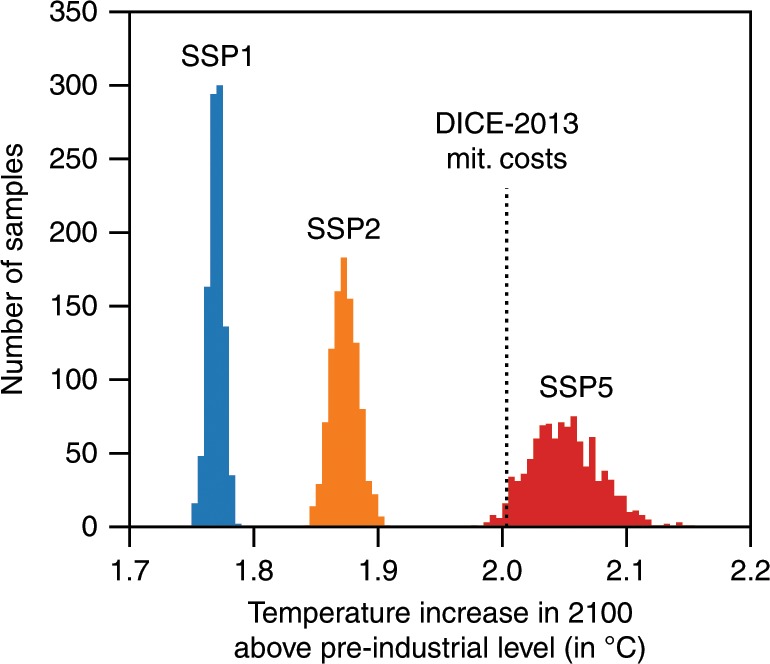
Economically optimal temperature increase for alternative mitigation-cost functions. The mitigation functions, which are sampled from the SSP fit, reflect different technological possibilities in the future as reflected by the SSPs. The dotted line shows the value for the benchmark estimate (DICE-2013).

Further tests also show robustness to alternative socioeconomic assumptions as described by the Shared Socioeconomic Pathways (SSPs)^25^ (Fig. 6). As the mitigation-cost function in DICE is strongly simplified, we investigate how our results change with functions that describe different technological possibilities in the future (Fig. 7). Similar to the differences between results for a range of damage functions, the uncertainty in mitigation costs reflects on the derived optimal warming level. Nevertheless, the mitigation costs deriving from the different SSPs tend to imply rather lower median optimal warming levels (1.8 °C, 1.9 °C, 2.0 °C).

## Discussion

Our findings build on the most recent empirical advances of impact estimates, which we consistently integrate in a dynamic IAM. These estimates are, however, not without critique, especially regarding the assumed functional relationship, the significance of using weather variables for insights into climate impacts and on other methodological challenges. In particular, using them in projections assumes that the historically observed temperature-impact link can be extrapolated into the future. Yet, this relation can change if further warming is associated with an unprecedented variation in climatic extremes for example with potential cascading effects^30–33^ or with the occurrence of devastating climatic tipping points^34,35^, or with significant changes in the societal response to warming. We also follow other studies using the estimates for projections^6,8^ to derive the benefits for smooth temperature paths without variability. The economic costs associated with temperature variability may, however, require even more stringent mitigation efforts.

Furthermore, assessing impacts in terms of GDP is an incomplete measure for the overall benefits of climate change mitigation as non-monetary losses such as loss of life and biodiversity are omitted. Unless adaptation to climate change becomes effective, most of these points suggest a strong underestimation of the mitigation efforts needed.

Similarly, a global analysis like ours, of course, neglects distributional issues as to who bears the burdens of damages as well as mitigation costs. Some specifications of the damage functions we employ here differentiate at least between two classes of income levels. Here, we have to make simplifying assumptions regarding shares of these classes to incorporate them into the one-region model, which constitute another source of uncertainty (Fig. 4). In general, a cost-benefit calculation has to be interpreted vary cautiously keeping ethical considerations in mind. Like other studies^36^ we use DICE as a parsimonious surrogate for more complex and spatially disaggregate IAMs. Future research should transfer our analysis to these IAMs to clarify questions of regional impact heterogeneity and to fully account for region-specific empirical estimates.

In our analysis, the leeway to reach the 2 °C target is considerably constraint by ruling out negative emissions in this century. Nonetheless, we show that, if future damages follow the same temperature dependence as historically observed, the overall damage costs will reach a level that renders 2 °C cost-benefit optimal. This result evolves as a direct consequence from the recently given empirical evidence attesting considerable marginal damage increases for higher temperatures and the universal functional behaviour of the mitigation costs in the vicinity of present-day temperatures (cf. Fig. 1).

## Methods

### The IAM DICE-2013

The IAM used for this analysis is DICE^16,20^, which fully couples a simple climate model with a Ramsey model of the global economy. DICE describes the interaction of climate change and economically optimal decisions of allocating the available income to consumption, to investment, and to mitigation efforts. Whereas consumption increases welfare to be maximized as the objective in the model, investment into production capital ensures future income. Production generating income thus assumes a crucial role for the well-being of present and future generations. The model also demonstrates the downside of increased production. If not mitigated, greenhouse gas emissions come as a by-product of economic activities. These gases accumulate in the atmosphere and drive—with some time delay—the global temperature. Climate impacts then cause economic losses that reduce the available income. Given all these trade-offs, the model searches for the allocation pathway that maximises welfare.

The DICE version we use is DICE2013Rv2_102213_vanilla_v24b.gms, which we abbreviate as DICE-2013 here and in the main text. This version was the most recent version when this research was started. Meanwhile, a more recent DICE version, i.e. DICE2016R-091916ap.gms, was released by William Nordhaus. These two versions are similar with respect to their analytical background^21^, but imply slightly different optimal temperatures (DICE-2016 implies a 0.2 °C higher optimal temperature occurring approximately 30 years later). DICE has been updated with respect to the calibration of the real gross domestic product (GDP), its future growth rates, population estimates, current emissions data, emission reduction costs, carbon intensity, the carbon cycle and the damage costs^21^.

In particular, the calibration of the carbon cycle has undergone significant modifications. As state-of-the-art climate models are too computationally expensive, simplified models that often consist of only a few linear equations are used in IAMs. However, it has been shown that many IAMs cannot fully reproduce the carbon cycle dynamics of complex, state-of-the-art models^37,38^. In particular, the linear carbon cycle representations reflect poorly the non-linear ocean response to higher atmospheric carbon levels^39^. Linear representations that are fitted to initial carbon uptake lead to too rapid removal of atmospheric CO_2_ after several decades^39^. Warming over the next centuries and the extent of necessary policy intervention are thus underestimated^37–39^. This problem also exists in DICE-2013, which is aimed to fit short-run carbon cycle dynamics (primarily the first hundred years)^21^ of larger models. Employing the carbon cycle model of DICE-2013 in this study thus means that although the model represents the carbon cycle dynamics in this century well (cf. Fig. 1 in Glotter et al.^39^ and the temperature development for the Representative Concentration Pathways in Supplementary Fig. 7), our results concerning the temperature target and the optimal policy efforts are rather conservative estimates. The error in the policy recommendations may become particularly large for small discount rates, which is important to recognize with respect to our robustness test with alternative preference parameter values (Fig. 5).

In DICE-2016, the linearity of the first-order differential equations is maintained, but the parameters are calibrated to give a good fit for the more distant future (periods up to 4000 years)^21^. The emission reduction costs have been adjusted slightly upwards in DICE-2016. Yet, this modification does not affect results significantly^21^.

As stated by Nordhaus^21^, the major change in DICE-2016 is the method for estimating the damage function. This adjustment, however, does not affect our analysis, as we replace the damage function by a new curve. Furthermore, as explained below, we use more recent estimates and projections to update DICE-2013. Given the nature and extent of the updates in DICE-2016, and our own recalibration efforts to incorporate recent data, we believe that using DICE-2013 as the basis model for our study is justifiable.

### Recalibrating DICE-2013

The original DICE-2013 simulation horizon starts with the year 2010. Instead of forcing the model to assume very low emission reduction efforts between 2010 and 2020, we make some minor modifications to have the simulation horizon start with the year 2020. To most parameters we assign their values in 2020 from the original model as the initial value. However, we assume a global average temperature increase above pre-industrial level of 1.2 °C by 2020^27^. We further use GDP projections from the World Economic Outlook by the IMF^40^ deflated to $US 2005 values (the base year for all values in DICE-2013). These values, together with the CO_2_-equivalent-emissions output ratio *σ* for 2020, imply industrial CO_2_ emissions of 37.52 GtCO_2_ in the year 2020. This number is slightly higher than 36.19 GtCO_2_ as projected for the RCP4.5 path^41^, but better reflects the latest observed increases in global emissions^42^. To update the cumulative industrial carbon emissions, we retrieve observed data from the PBL 2017 report^42^ and interpolate linearly between the last observation in 2016 and the projected emissions in 2020 to obtain emissions for 2017–2019. Using the updated GDP value in 2020, we also adjust the value of the initial production capital. For consistency with the estimated impacts, we recalibrate the 5-year-period DICE-2013 to an annual time step version with 600 years model run time in total.

### The temperature–growth relation

The costs of warming are often given in terms of the contemporaneous changes in GDP^43–45^. This static approach, however, omits dynamic effects like changes in investment through which climate change may affect economic growth and hence future GDP^46^.

An early estimate by Dell et al.^4^ (DJO, hereafter) of the temperature–growth link finds a linear relation between growth and temperature. It also shows that only poor countries suffer from temperature. The results for rich countries are not significant and are inconclusive about whether these countries benefit or suffer from warming.

BHM have updated this estimation by finding evidence for a non-linear, quadratic relation, which they attribute to the longer dataset they use. BHM also argue that the inconclusive results concerning the rich countries’ impacts stem from the linear relation found. According to the BHM estimates, the poor countries are located on the downward facing slope of the concave relation between temperature and growth. In contrast, rich countries are distributed around the optimum of this function. A linear regression translates this relation into (inconclusive) statements that the rich countries are not vulnerable at all, or depending on the exact specification, might be affected slightly in a positive or negative way.

The differences in these estimation results lead to completely different interpretations. While DJO predict that all countries have a vulnerability that decreases over time as they become richer, BHM observe that countries get increasingly vulnerable over time as they become warmer on average.

DJO’s estimates have accelerated research about how to model growth impacts for climate policy assessment. The channels through which climate change can affect economic development are manifold. Apart from direct production reductions that trigger higher-order effects such as reduced investment and thus alter the economic growth dynamics, climate change may also affect the progress of research and slow the growth of total factor productivity (TFP) or accelerate depreciation of the capital stock.

Moore and Diaz^47^ investigate the two latter pathways individually in a two-region DICE model. They choose to apply the DJO estimates that result for a lagged response of the damage costs to temperature. For this specification, the estimated negative temperature–growth relations for poor countries are significant at the 10 percent level and not significant for rich countries. To implement the point estimates, Moore and Diaz apply constant scaling factors to the TFP growth rate to reproduce the total estimated economic effects. Just as our study, Moore and Diaz^47^ find that optimal policy stabilizes global warming at 2 °C.

Based on DJO’s estimation method, Lemoine and Kapnick^7^ develop probability distributions for regional economic impacts of future climate change by combining distributions for the historical temperature–growth link with SSPs^25^ and global climate model results. Similar to Moore and Diaz^47^, they transfer their estimates to DICE-2013 by an explicit model of TFP growth reductions.

Dietz and Stern^48^ include more than one impact channel of growth reductions and also find that optimal emission reduction efforts must be significantly increased. As empirical studies have so far not been able to quantify by how much the observed growth reductions can be traced back to the potential channels, studies including more than one impact channel have to rely on mostly arbitrary assumptions about the contribution of the channels to the growth reductions.

Guivarch and Pottier^49^ investigate whether certain damage structures, e.g. those that imply that only TFP growth is affected, lead to a higher social cost of carbon than damage on production itself. They find that if the overall damage magnitude is the same, the ranking between these alternative models is not unequivocal and rather depends on the choice of the preference parameters.

In the absence of a comprehensive and empirically validated model that captures the growth effects, we limit ourselves to finding a production reduction function, i.e. a damage function, that leads to the same growth effects as estimated. Our damage function thus serves to emulate the estimated growth impacts, without attempting to capture the underlying mechanisms. Compared to Moore and Diaz^47^ and Dietz and Stern^48^ ours is an alternative approach that does not require making any arbitrary assumptions. We believe that our approach adds substance to the literature concerned with developing damage functions for IAMs^43–45^. These damage function often lack recent empirical evidence, in particular with respect to the growth impacts^13^.

As opposed to Moore and Diaz^47^ and Lemoine and Kapnick^7^, the estimates we use for the damage function development stem from the more recent empirical work in the BHM study, which accounts for a non-linear temperature–growth relation.

The BHM estimates have initiated a necessary debate about possible methodological advances to estimate the growth impacts, in particular with respect to the assumed functional relationship^50^, the significance of using weather variables for insights into climate impacts^11,51^ and on other methodological challenges^9^. Even though only short time series and small increases in temperature and other weather variables^52^ are available for estimation, enriching cost-benefit analysis of climate policy with the currently existing empirical evidence about the impacts is a necessary and highly relevant improvement to be made^13^.

As also stated in the main text, the implications of future damages evolving according to the BHM estimates have been investigated so far by using predetermined scenarios of warming and economic growth. An important contribution by Ricke et al.^9^ finds that the BHM estimates are associated with a rather high social cost of carbon, which may indicate that optimal policy should be stringent. Burke et al.^8^ show that there is a large potential damage reduction if temperature increase is limited to 1.5 °C or 2 °C. Ueckerdt et al.^10^ additionally account for the costs of mitigation in a model with exogenous economic growth and temperature development. Compared to these contributions, our method of developing a damage function from the BHM estimates within an IAM allows maintaining the diverse feedback processes between the economic and climate mechanisms given by the DICE model.

### The temperature-induced growth impacts according to BHM

Here, we give a short summary of the estimation of the relation between temperature and economic growth on which we base our analysis (for more details please see Burke et al.^6^ and the associated supplementary material).

Burke et al.^6,8^ estimate this relation for all countries in the world based on observed data from 1960–2010 based on the statistic model

1 $${\Delta {\mathrm{ln}}\left( {\frac{{Y(n,t)}}{{L(n,t)}}} \right)} = {h\left( {T^{{\mathrm{ATM}}}\left( {n,t} \right)} \right) + \lambda _1P\left( {n,t} \right) + \lambda _2P\left( {n,t} \right)^2} \,\,\,\,\,\,\,\,\\ + \,{\mu \left( n \right) + \nu \left( t \right) + \theta \left( n \right)t + \theta _2\left( n \right)t^2 + {\it{\epsilon }}\left( {n,t} \right)}$$

for all countries *n* and considered years *t*. The dependent variables are the first differences of the natural logarithm of annual real (inflation-adjusted) GDP per capita (being the fraction of GDP *Y* and population *L*). These first differences are interpreted as annual growth rates of income. The independent variables are functional specifications *h* of the absolute average regional temperature *T*^ATM^ and precipitation *P*. Furthermore, time-invariant factors, e.g. history and topography, are accounted for by including country-specific fixed effects *μ*. Time-varying factors including abrupt shocks, e.g. global recessions and shocks to energy markets, and slowly evolving changes, e.g. demographic shifts and evolving institutions, are captured by year-specific fixed effects *ν*(*t*) and country-specific time trends $\theta \left( n \right)t + \theta _2\left( n \right)t^2$, respectively.

Burke et al.^6,8^ find strong evidence for a global quadratic temperature response according to

2 $$h\left( {T^{{\mathrm{ATM}}}\left( {n,t} \right)} \right) = \beta _1T^{{\mathrm{ATM}}}\left( {n,t} \right) + \beta _2\left( {T^{{\mathrm{ATM}}}\left( {n,t} \right)} \right)^2.$$

They also test specifications of *h* with different functional temperature response and find no improvements in the performance of these alternative models.

For the global sample, Burke et al.^8^ find statistically significant estimates for the parameters in the temperature response function of *β*_1_ = 0.0127 and *β*_2_ = −0.0005 (Extended Data Table 1 in Burke et al.). While the country-level estimates given by Burke et al. would require population weights for usage in a global IAM, the global estimates can be used in a global IAM directly. These values, thus, constitute our base calibration.

Burke et al.^6^ also compare data from 1960 to 1989 with data from 1990 to 2010 and find that the response has not changed significantly over time. This indicates that adaptation processes that could have changed the response in the past are not observable in the data. Furthermore, it implies that the investment response to current or future climate change, which affects economic growth, has not altered qualitatively over time despite increased availability of information about the climate problem.

In a global analysis, Burke et al.^6,8^ extrapolate the estimated impact relation into the future and derive projections of future levels of income per capita relative to a world with temperatures fixed at their 1980–2010 average. In particular, the evolution of the global income per capita is described as

3 $$\frac{{Y(t + 1)}}{{L(t + 1)}} = \frac{{Y(t)}}{{L(t)}}\left( {1 + \eta (t) + \phi (t)} \right).$$

Here, *η* is the hypothetical growth rate in the absence of climate change and *ϕ*(*t*) the additional effect of warming on growth in that year *t*. The growth rate *ϕ*(*t*) is expressed in terms of the estimated response function *h* as

4 $$\phi (t) = h\left( {T^{{\mathrm{ATM}}}(t)} \right) - h\left( {\bar T^{{\mathrm{ATM}}}} \right),$$

with $T^{{\mathrm{ATM}}}(t)$ being the global absolute temperature in a year *t* and $\bar T^{{\mathrm{ATM}}}$ being the average 1980–2010 temperature. This average temperature represents climatic conditions to which the global economy and society have grown accustomed to and which are assumed to have no economic effects.

### Deriving a new damage cost function for DICE

The climate impact function *ϕ*(*t*) is not tantamount to damage functions which are usually employed in IAMs. These damage functions typically describe reductions of the GDP level, which can be perceived as a productivity reduction of labour and capital. This can be seen when extending the standard Cobb-Douglas production function by temperature sensitive labour productivity $A^{\mathrm{L}}\left( {T^{{\mathrm{ATM}}}\left( t \right)} \right)$ and temperature sensitive capital productivity $A^{\mathrm{K}}\left( {T^{{\mathrm{ATM}}}\left( t \right)} \right)$ as follows:^6^

5 $$\begin{array}{*{20}{l}} {Y(t)} \hfill & = \hfill & {A(t)\left( {A^{\mathrm{K}}\left( {T^{{\mathrm{ATM}}}\left( t \right)} \right)K(t)} \right)^\gamma \left( {A^{\mathrm{L}}\left( {T^{{\mathrm{ATM}}}\left( t \right)} \right)L(t)} \right)^{1 - \gamma }} \hfill \\ {} \hfill & = \hfill & {\underbrace {A^{\mathrm{K}}\left( {T^{{\mathrm{ATM}}}(t)} \right)^\gamma A^{\mathrm{L}}\left( {T^{{\mathrm{ATM}}}(t)} \right)^{1 - \gamma }}_{ = f\left( {T^{{\mathrm{ATM}}}(t)} \right)}A\left( t \right)K\left( t \right)^\gamma L(t)^{1 - \gamma }} \hfill \\ {} \hfill & = \hfill & {f\left( {T^{{\mathrm{ATM}}}\left( t \right)} \right)Y^{{\mathrm{gross}}}\left( t \right),} \hfill \end{array}$$

with GDP gross of level effects *Y*^gross^(*t*), temperature-independent TFP *A*(*t*), productive capital *K*(*t*), labour *L*(*t*), output elasticity of capital *γ* and temperature sensitive productivity $f\left( {T^{{\mathrm{ATM}}}(t)} \right)$. GDP net of level damage costs *Y*(*t*) corresponds to the observed income levels in Eq. (3).

Unlike a level damage function $f\left( {T^{{\mathrm{ATM}}}(t)} \right)$, the climate impact function *ϕ*(*t*) is part of the GDP growth rate and thus entangles level effects and the investment response leading to growth effects. Directly using the growth rate *ϕ*(*t*) together with Eq. (3) in DICE would result in an exogenous growth model, i.e. in a model in which investment is predetermined and cannot be adjusted optimally. To maintain the growth model feature, we seek a damage function $f\left( {T^{{\mathrm{ATM}}}(t)} \right)$ as in Eq. (5) that is—together with the growth effects triggered by investment—consistent with the estimated growth impacts.

To this end, we first convert the temperature increase ${\mathrm{\Delta }}T^{{\mathrm{ATM}}}(t)$ (in °C from 1900) computed by the climate module in DICE to the absolute annual temperature $T^{{\mathrm{ATM}}}\left( t \right)$ in the estimated response function *h* according to

6 $$T^{{\mathrm{ATM}}}\left( t \right) = T_{2010}^{{\mathrm{ATM}}} + {\mathrm{\Delta }}T^{{\mathrm{ATM}}}\left( t \right) - {\mathrm{\Delta }}T_{2010}^{{\mathrm{ATM}}},$$

with the absolute global temperature in the year 2010 $T_{2010}^{{\mathrm{ATM}}}$ and the global average temperature increase in 2010, ${\mathrm{\Delta }}T_{2010}^{{\mathrm{ATM}}}.$ For 2010, we use the average temperature over 2005–2010 to calibrate $T_{2010}^{{\mathrm{ATM}}}$. The data for calibration is compiled from a NASA dataset^53,54^. The global average temperature increase in 2010, ${\mathrm{\Delta }}T_{2010}^{{\mathrm{ATM}}}$, stems from the original DICE-2013 version. Important for the choice of the reference year, here 2010, is the availability of the required temperature data. Apart from that, the reference year can be chosen arbitrarily.

To derive a damage function *f* consistent with the impact estimates, we use an iterative algorithm that allows disentangling the productivity loss function as described by Eq. (5) from the investment response, both of which jointly cause the growth impact *ϕ*(*t*). Extrapolating the past relation between temperature increase and productivity losses into the future is only a valid approach if the future economy and its vulnerability are similar as in the past. To obtain a scenario that emulates such a future world, we impose three key assumptions on the calibration run.

First, we exclude the option to reduce emissions and thus mimic the absence of any notable emission reduction efforts from 1980–2010. Growth effects that might be induced by reallocating investment resources to mitigation efforts can thus be abstracted from.

Second, as the estimated response relation for the years 1960–1989 does not differ significantly from the estimations for the years 1990–2010, notable adaptation is not observable in the data^6^. Accordingly, we also abstract from adaptation as a policy tool. Similar to the first assumption, this means that growth effects that might have resulted from reallocating investment resources to adaptation can be ignored.

Third, we assume that investment is not slowed down to reduce emissions in the absence of mitigation efforts. Yet, the investment decision takes into account the emergence of future productivity losses making investments less profitable over time. Hence, investment reacts to productivity losses, but it is not used for damage-cost reduction.

Essentially, the third assumption is equivalent to postulating that the investment decision is made under ignorance of the temperature-productivity nexus. Accordingly, in the calibration run we seek a time series *f*(*t*), rather than a temperature-dependent function, that fulfils

7 $$f\left( {t + 1} \right)\frac{{Y^{{\mathrm{gross}}}\left( {t + 1} \right)}}{{L\left( {t + 1} \right)}} = \frac{{Y\left( t \right)}}{{L\left( t \right)}}\left( {1 + \eta \left( t \right) + \phi \left( t \right)} \right).$$

For the initial period we approximate $f(1) \approx \left( {1 + \phi (1)} \right)$ with *ϕ*(*t*) resulting from Eq. (4) with the initial absolute temperature $T^{{\mathrm{ATM}}}\left( 1 \right)$ from Eq. (6).

Preceding the iteration, we solve the model with no climate damage costs to obtain the investment rate $s_t^{{\mathrm{nocc}}}$ optimal in absence of climate change.

The iteration is then performed over a set of functions $f(t)^{\left( j \right)}$ with *j* being the number of iteration steps. Starting with $f(t)^{\left( 1 \right)} = 1$, i.e. with zero climate damage for all temperatures, the iteration (Supplementary Fig. 8) encompasses the following steps:

First, solving DICE with a damage function $f(t)^{(j)}$: We solve the model with $f(t)^{(j)}$ as the damage function, yielding time series of income $Y^{{\mathrm{gross}}}(t)^{(j)}$ and $Y(t)^{(j)}$ as well as $\phi \left( t \right)^{(j)}$ that evolves from Eq. (4). Applying the investment rate $s_t^{{\mathrm{nocc}}}$ to $Y(t)_{}^{\left( j \right)}$ provides the hypothetical growth rate $\eta \left( t \right)^{(j)}$. Evaluating Eq. (7) with $f(t + 1)_{}^{\left( j \right)}$, $Y^{{\mathrm{gross}}}(t + 1)_{}^{\left( j \right)}$, $Y(t)_{}^{\left( j \right)}$, and $\eta \left( t \right)^{(j)}$ we obtain the actual effect $\bar \phi (n,t)^{(j)}$ of the temperature time series on growth in iteration step *j*. This growth rate, which is crucially influenced by the assumed function $f(t)_{}^{\left( j \right)}$ and the associated investment response, is sought to converge towards the estimated temperature–dependant time series $\phi \left( t \right)^{(j)}$ given by Eq. (4). Thus, the iteration algorithm is stopped once the time-average absolute deviation between the two rates $\bar \phi$ and *ϕ* has become sufficiently small, here, less than 6 × 10^−5^. At the same time, all other time series, in particular the investment response, the temperature time series and the damage time series, converge.

Second, updating the damage function for the next iteration step: To derive $f(t)_{}^{\left( {j + 1} \right)}$ to be used in the next iteration step, we again employ Eq. (7). Unlike in the first iteration step, we now compute the function values of *f*(*t* + 1) that fulfil Eq. (7) for the time series $Y^{{\mathrm{gross}}}(t)_{}^{\left( j \right)}$, $Y(t)_{}^{\left( j \right)}$, and $\eta \left( t \right)^{(j)}$ using the estimated temperature-induced growth rates $\phi \left( t \right)^{(j)}$ that evolve from Eq. (4). We use the resulting time series, which we refer to as $\tilde f(t)$, to update the damage function for the next iteration step according to

8 $$f(t)^{(j + 1)} = f(t)^{(j)} + \frac{{\tilde f(t) - f(t)^{(j)}}}{2}.$$

The time series $f(t)^{\left( {j_{{\mathrm{last}}}} \right)}$ of the last iteration defines the damage function that generates—together with the investment response—the growth impacts estimated. For the derivation of this function, it was postulated that the investment decision is made under ignorance of the temperature–productivity nexus. This assumption necessitates seeking a time series rather than a temperature-dependent function. For the simulation runs, however, we return to the original narrative of the damage function in DICE. Accordingly, the notable difference between the damage calibration run and the simulation runs is that the optimal decisions now fully incorporate the information about the future climate damage costs. In particular, the investment decision accounts for the costs that this investment eventually causes, which requires having a temperature-dependent function. The temperature dependence is crucial for choosing the optimal temperature path. We therefore tie together the information given by the time series $f(t)_{}^{\left( {j_{{\mathrm{last}}}} \right)}$ with the temperature increase ${\mathrm{\Delta }}T(t)_{}^{{\mathrm{ATM}},(j_{{\mathrm{last}}})}$ of the same iteration run. We do so by expressing that the damage $f(t)_{}^{(j_{{\mathrm{last}}})}$ observed in the iteration run is caused by the temperature increase ${\mathrm{\Delta }}T(t)_{}^{{\mathrm{ATM}},(j_{{\mathrm{last}}})}$ at that time. If, for instance, in the year 2030 a damage of 10% is caused and in the same year the temperature increases by 1.5 °C, then the temperature-dependent function conveys the information that a temperature increase of 1.5 °C implies damage costs of 10%, regardless of the timing. Accordingly, if the 1.5 °C warming occurs at a different point in time in the simulation runs than in the damage calibration run, then it is still associated with a 10% loss. This means that the damage function does not reproduce BHM’s growth estimates for any other scenario than the calibration run that emulates the conditions for which extrapolation of the estimates is justifiable.

In short, for each time step *t*, $t = 1, \ldots ,600$, we specify $f\left( {{\mathrm{\Delta }}T_{}^{{\mathrm{ATM}}}} \right)$ by $f( {{\mathrm{\Delta }}T(t)^{{\mathrm{ATM}}}} ): = f(t)^{(j_{{\mathrm{last}}})}$, i.e. the function value of $f(t)_{}^{(j_{{\mathrm{last}}})}$ is now defined in ${\mathrm{\Delta }}T_{}^{{\mathrm{ATM}}}$ and not in *t.* Just as $f(t)_{}^{(j_{{\mathrm{last}}})}$, this function is discrete in 600 points. In the simulation runs, we interpolate this function linearly between these points. This procedure has the advantage that we do not have to make any assumptions, as opposed to approximation which would require prescribing a functional form of the approximated function, and do not lose the iteratively obtained precision. Furthermore, the function is interpolated between a sufficient number of points to maintain the non-linearity of the function despite the linear interpolation. This new function then replaces the damage cost function in the policy runs.

### Robustness of results

In the following, we subject our results to extensive robustness tests. First, we add to the climate sensitivity analysis from the main text by accounting for an entire probability density function for the ECS values. Second, we examine the implications of uncertainty in BHM’s estimations. In this respect we account for alternative estimates of *β*_1_ and *β*_2_ on the one hand and different model specifications on the other hand. This analysis is followed by a comparison with the DJO estimates. Third, we investigate the influence of uncertainty about the socioeconomic future by recalibrating the DICE model according to a selected set of SSPs. As a by-product of this calibration, we obtain mitigation-cost functions that emulate the costs from a detailed process model and thus represent another advancement of the DICE model. The derivation of these functions allows us to test the sensitivity of our results with respect to these alternative costs of emission reduction. We complete this section by giving more information on the robustness test with respect to the preference parameters shown in the main text.

### Robustness with respect to ECS

Here, we extend the uncertainty analysis with respect to the ECS values as shown in Fig. 1. To this end, we employ a probability distribution of ECS values that was estimated from a suite of GCM simulations (cf. Figure 3 (A) in Roe and Baker^26^ and Supplementary Fig. 9).

The resulting distribution of economically optimal temperatures in 2100 inherits properties from the ECS probability distribution. As also shown in the main text, higher ECS values imply a higher temperature target due to the limited leeway to reach lower temperatures with climate policy. Furthermore, the more detailed sensitivity analysis confirms that the most likely temperature targets lie around 2 °C. Yet, there is a certain, albeit very small, chance that the economically optimal temperature target might be significantly higher, maybe up to 4 °C. The likelihood for these high targets however decreases considerably for all ECS values beyond 4 °C. Accordingly, the tail probabilities of the high ECS values are passed on to the distribution of the optimal temperatures in 2100.

### Robustness with respect to the estimated damage function

To quantify uncertainty in the estimates of *β*_1_ and *β*_2_ in Eq. (2), Burke et al.^8^ implement bootstrapping strategies which are based on sampling by country, by year and by five-year blocks. They sample by country by drawing with replacement from their list of 165 countries a total of 165 countries and re-estimate the response function with that set. This sampling method allows for correlation in residuals within countries over time. Likewise, they sample over the years and the 5-year blocks, which allows for cross-sectional correlation in residuals in a given year and for both temporal and cross-sectional dependence in residuals, respectively.

We use these three methods for our analysis of uncertainty in the estimated response function. For each bootstrapping strategy, we draw 1000 samples. For each sample we derive the estimates for *β*_1_ and *β*_2_, apply the iteration over the damage functions for the new response function *h* and use the resulting function in the policy runs. The results for the three different bootstrapping strategies are illustrated in Fig. 3 and Supplementary Figs. 1 and 2, respectively.

Despite the substantial uncertainty in the impact estimates, 40% of the ensemble runs for ECS of 2.9 °C show an optimal warming below 2 °C (Supplementary Fig. 10). This share increases steeply for slightly higher warming targets. None of the damage-cost curves implies 2 °C as economically optimal for ECS of 4 °C. For ECS of 2 °C as many as 63% of the uncertainty ensemble results comply with the 2 °C target.

In addition, we investigate the sensitivity of our results to BHM’s model specification (Fig. 4). The main BHM specification, which is also the main specification in our study, does neither account for the possibility of an economic response that is lagged in temperature, nor does it differentiate between responses with respect to income levels. BHM tested these alternative specifications with the following results.

Pooled long-run specification: The test with lagged terms shows that the response on pooled, or global, GDP becomes substantially more negative, because cooler regions no longer unambiguously profit from warming. However, as accounting for more lags renders the estimation more uncertain, BHM reject neither the hypothesis of a short-run, or instantaneous, temperature effect nor the hypothesis of a long-run, or lagged, response.

Differentiated short-run specification: As pioneering work by DJO indicates that the income level is the determining factor of the impact on GDP, BHM also re-estimate the response for rich and poor countries separately. The optimum of the poor-country response function is observed to occur for a higher temperature than for the pooled, global sample. Accordingly, the cumulative response is smaller than in the main specification. While the rich countries’ response is found to be significantly different from zero, the parameter adjustment made for poor countries, however, is not significant. Accordingly, in contrast to DJO, BHM cannot reject the hypothesis that rich and poor countries have the same response function.

Differentiated long-run specification: BHM also test a model that accounts for lagged effects and distinguishes between rich and poor countries. Just as in the differentiated short-run specification, differentiating with respect to income renders the cumulative response smaller than for the pooled long-run response function. However, splitting the sample in rich and poor countries as well as accounting for additional uncertain parameters to capture the long-run effects produces an overall large projection uncertainty.

We expect that these outcomes will be largely reflected in our results. We use their bootstrapped estimation results to test the sensitivity of our results to these alternative models.

For this purpose, we expand Eq. (2) by the corresponding terms describing the lags and/or the GDP share of rich and poor countries. The GDP share is modelled as a linearly decreasing function as described in detail below. As also argued there, the differentiation with respect to the income level would preferably require a two-region model. With a global IAM we instead try to generate a damage function that aggregates over the different impacts for rich and poor countries. We thus make assumptions about the poor’s share in the global GDP. While this modelling certainly is a makeshift solution, it serves to provide some impression of how the different vulnerabilities affect the optimal solution. Our tests with different specifications for the poor countries’ share in global GDP show only marginal changes in the results, as the poor countries’ GDP losses are small in absolute numbers for all specifications. The only exception to this is the case in which the poor countries’ GDP share increases significantly. As so far this share in global GDP has been observed to decrease over time, we believe that our assumption of a linearly decreasing share is feasible.

As expected, the implied optimal end-of-century temperatures for the different model specifications reflect the findings by BHM (Fig. 4 and Supplementary Figs. 3–5). As shown by BHM the differentiated short-run specification implies less severe losses. Accordingly our results reflect that the economically optimal end-of-century temperatures turn out to be higher. By contrast, the other two specifications, which are associated with higher damage costs, imply that mitigation efforts are to be strengthened further. For a 2.9 °C climate sensitivity, limiting temperature increase to well below 2 °C is optimal under these model specifications.

Although based on the same dataset, different model specifications can imply significant discrepancies in the estimates. As the damage cost estimates are of major importance for the optimal policy solution in IAMs, it is not surprising that our results are sensitive to these model specifications. However, as three out of four model specifications imply an economically optimal temperature target of 2 °C or even lower for a 2.9 °C climate sensitivity, we consider our results relatively robust to the different BHM model specifications.

### Comparison with the DJO estimate

As described above, the pioneering work by DJO describes the relation between temperature and growth to be linear and reveals that only poor countries suffer from temperature. However, the results for rich countries are not statically significant with point estimates ranging from slightly positive in the zero-lag specification to slightly negative in the 5-lag specification.

While our study is based on the more recent BHM estimates, which exhibit a non-linear relation between temperature increase and economic growth, we test here whether our results concerning the end-of-century optimal temperature might also hold for the DJO estimation results.

As indicated above, the different specifications of the DJO regressions might lead to very different results. So far, studies have used different specifications of the DJO regression and do not agree on the question of whether the estimates for the rich countries hold sufficient informative value to be used for analysis. For instance, Moore and Diaz^47^ employ the estimates for the 10-lag specification that gives a negative relation between temperature and growth for rich countries. Ricke et al.^9^ include the 0-lag specification and ignore the positive impact relation for the rich countries. For a complete picture, we here show the results for all lag specifications given by DJO with and without rich countries’ impacts (Fig. 4 and Supplementary Fig. 6).

This analysis, however, must be treated with caution. Our study’s aim is to generate a damage function for a global IAM as DICE. The implementation of DJO’s estimates, however, requires model with at least two regions, preferably with implemented welfare weighting in the optimization. Nevertheless, to get a rough impression of the implications of DJO estimates for our results with DICE, we impose assumptions about the share of the poor countries’ GDP in the global GDP.

To implement the DJO estimates, we change *ϕ*(*t*) in Eq. (3) to $\phi \left( t \right) = \delta \Delta T(t + 1)^{{\mathrm{ATM}}}$ and let the coefficient $\delta$ differ for rich and poor countries. In addition, as DICE cannot track how many countries are poor or rich, we impose assumptions about the share $\varsigma (t)$ of the poor countries’ GDP in the global GDP. With this share, we can extend Eq. (3) to

9 $$\frac{{Y(t + 1)}}{{L(t + 1)}}L\left( t \right) = \, {\upvarsigma}\left( t \right)Y\left( t \right)\left( {1 + \eta \left( t \right) + \phi \left( t \right)^{{\mathrm{poor}}}} \right) \,\,\,\,\,\,\,\,\,\,\,\,\,\,\,\,\,\,\,\, \\ + \,\left( {1 - {\upvarsigma}\left( t \right)} \right)Y\left( t \right)\big( {1 + \eta \left( t \right) + \phi \left( t \right)^{{\mathrm{rich}}}} \big)$$

with the $\phi \left( t \right)^{{\mathrm{poor}}}$ and $\phi \left( t \right)^{{\mathrm{rich}}}$ describing the alternative specifications of *ϕ*(*t*) for poor and rich countries, respectively. This growth equation partitions global GDP into poor and rich countries’ GDP and thus acts as a makeshift to get a rough idea of the effects in a two-region model with sophisticated welfare weighting^47^.

We assume that ${\upvarsigma}\left( t \right)$ decreases linearly with global GDP per capita. For poverty defined as in DJO, that is having a below-median PPP-adjusted per capita GDP in the first year the country enters the dataset, this development is observable in the data^40^ of the past decades. As ${\upvarsigma}\left( t \right)$ only makes a statement about the poor countries’ relative contribution to global GDP, differing narratives about the future world are reconcilable with our modelling choice. For instance, rising global prosperity might be associated with increasingly many countries overcoming their poverty and assuming a rich countries’ vulnerability. Alternatively, rich countries could get even richer, while the poor countries do not prosper at all or get even poorer.

We calibrate the linear function ${\upvarsigma}\left( t \right)$ using data from 1980^40^ and employing the assumption that the largest GDP per capita value in the absence of climate change as computed by DICE leads to ${\upvarsigma} = 0.$ Hence, although the GDP share of poor countries declines, we assume that poverty will never be fully eradicated over many decades.

A linearly decreasing ${\upvarsigma}\left( t \right)$ implies that if global prosperity increases, ceteris paribus, global GDP becomes less sensitive to temperature. We contrast this simulation with a scenario, in which the value for 1980 does not decline, i.e. we assume ${\upvarsigma}\left( {\mathrm{t}} \right) = {\upvarsigma}^{1980} \approx 0.1272$.

The alternative specifications of DJO we test here do not imply results that are virtually different with respect to the assumed ${\upvarsigma}\left( {\mathrm{t}} \right)$ (Supplementary Fig. 6). The reason for this is that the poor countries’ contribution is small for both specifications for ${\upvarsigma}\left( {\mathrm{t}} \right)$. Yet, this means that the treatment of the rich countries estimated impacts matter more. In the 0-lag and the 1-lag specification, the major share of GDP generated by rich countries is positively affected by warming. In this case, the BAU end-of-century temperature is optimal. In contrast, excluding the non-significant estimation results from the damage calibration leads to optimal temperatures that are only slightly higher than for the BHM model. A different situation arises for the 5- and 10-lag specification. Including the negative impact relation for the rich countries indicates optimality of significantly lower temperatures than for the BHM estimates. The DJO estimates thus imply largely differing results, ranging from 1.7 °C to 4 °C optimal warming. Most results, however, lay in a range between 1.7 °C and 2.3 °C (Fig. 4).

### Uncertainty with respect to alternative socioeconomic futures

In this section, we investigate the sensitivity of our results to alternative assumptions about the socioeconomic future. To facilitate the analysis of socioeconomically determined vulnerabilities, the SSPs were developed to describe possible future developments that together result in differing challenges for mitigation and adaptation^55^.

In DICE, these narratives are reflected by the developments of the population size, the TFP, carbon intensity, the mitigation costs, and the capital elasticity describing the division of income between capital and labour. Here, we limit our sensitivity study to a selected set of SSPs, i.e. SSP1 (Sustainability—Taking the Green Road), SSP2 (Middle of the Road) and SSP5 (Fossil Fuelled Development) to obtain a good impression of how alternative challenges for emission reduction affect the cost-benefit-optimal results. We ignore SSP3 (Regional Rivalry) and SSP4 (Inequality) as we believe that the problems induced by the depicted increasing regional fragmentation and the resulting obstacles for adaptation deserve a more explicit modelling than it is currently the case in DICE.

To recalibrate DICE according to these SSPs, we use data (until 2100) of the integrated energy-land-economy-climate scenarios generated by the REMIND-MAgPIE model^56^. REMIND-MAgPIE belongs to the IAMs with a detailed description of the energy sector that were chosen to translate the SSP narratives into quantitative projections^25^. As a result of the interpretation process of the narratives and the different model designs, each IAM model features alternative interpretations of the SSPs. For each SSP, a different IAM was selected to generate the so-called Marker Scenario. For our calibration exercise, we do not draw on the simulation output from the different marker models, but opt to rely on the data generated by only one model to avoid compatibility issues. So far, SSP1, SSP2, and SSP5 have been examined with REMIND. The scenarios computed consist of baselines in which climate policy is absent and of runs in which mitigation efforts comply with the Representative Concentration Pathways (RCPs). For this, a new, intermediate RCP of 3.4 W m^−2^ was developed due to its importance for exploring the attainability of the 2 °C target^25^.

We adopt the given population time series and keep the population constant after 2100. While this assumption certainly is far from realistic, it serves to distinguish the different scenarios in terms of different population sizes. We follow Leimbach et al.^57^ by assuming a capital elasticity of 0.35 for SSP1 and SSP2 and a higher value of 0.45 for SSP5. We also adopt their assumed capital price level (return rate on gross capital Investments) of 0.12 for all SSPs to compute the initial capital level (cf. Leimbach et al.^57^). Together with the baseline GDP time series we use this new parametrization to derive a matching TFP time series in the Ramsey model without climate change. We then employ this time series to fit the parameters describing the TFP development in DICE (Fig. 6b). We also recalibrate the DICE mitigation-cost parameters using the mitigation costs from the SSP scenarios. The mitigation costs in REMIND-MAgPIE equal the reduction of GDP with respect to the baseline case^58^. The carbon intensity needed for this fit and for the scenario runs results from dividing the baseline emissions by the baseline GDP. In contrast to the original mitigation-cost function in DICE, the resulting mitigation functions are thus calibrated against a detailed process model (Fig. 6c).

The socioeconomic conditions described by SSP1 and SSP2 leave sufficient leeway to aim for optimal end-of-century temperatures well below 2 °C (Fig. 6a). By contrast, the fossil-fuelled development portrayed by SSP5 renders successful climate policy much more difficult and implies optimal end-of-century temperatures around 2.5 °C.

As we have calibrated the mitigation-cost functions to simulations in which negative-emission technologies are employed, we also test the sensitivity of our results with respect to the availability of negative-emission technologies in this century. To simplify matters, we assume that the potential availability does not increase over time. However, the full mitigation potential is not assumed instantaneously in our simulations, rather increases over time. These simulations show that it is optimal to harness the increased mitigation potential to further reduce temperatures at the end of the century (Supplementary Fig. 6d).

The socioeconomic conditions in the future certainly play an important role for optimal policy design, yet they do not alter the message that mitigation efforts should be very stringent to come close or even lower 2 °C at the end of the century. The reason for this is the magnitude of the potential damage costs for higher temperatures.

### Sensitivity to alternative mitigation costs

The modelling of mitigation processes in DICE is often considered to be too simple^47^, because the cost function is not calibrated against a detailed process model, there is no expansion constraint for emission reduction^59,60^, and it does not affect factors of production or TFP^61^.

Here, it is not our intention to tackle these deficiencies. Rather, we aim to examine the sensitivity of our results to alternative mitigation-cost functions. For this, we leave the original socioeconomic setting of our DICE model unchanged and implement the three mitigation-cost functions that we recalibrated against a process model for the SSP sensitivity analysis (see above). Furthermore, we control for uncertainty in our calibration procedure. We do so by using the variance of the parameter estimate and the estimated optimal value to derive normal distributions for each parameter and each SSP. From each of these distributions we sample 1000 sets of parameters, i.e. 1000 alternative mitigation-cost functions.

We find that the Paris Agreement is also cost-benefit optimal when assuming these three mitigation-cost functions (Fig. 7). The spread in the results for each SSP is rather small, showing that potential errors in the fit are negligible for the results.

The reason for this high robustness with respect to the mitigation costs are the significant marginal damage increases for higher temperatures and the universal functional behaviour of the mitigation costs in the vicinity of present-day temperatures (cf. Fig. 1).

### Background information on the social preferences

The preferences as displayed in Fig. 5 are represented by the IRSTP and the elasticity of the marginal utility of consumption. The initial rate of social time preference *ρ* is used to assign different weight to the utility *U* of per capita consumption $c_t = \frac{{C_t}}{{L_t}}$ at different time points $t \in \left[ {1,T} \right]$ in the overall welfare function. In DICE, this social-welfare function *W* is given by

10 $$W = \mathop {\sum }\limits_{t = 1}^T \left( {\frac{1}{{1 + \rho }}} \right)^{t - 1}L_tU\left( {c_t} \right).$$

In other words, *ρ* relates to impatience in consumption; a higher IRSTP gives more emphasis to present rather than to future utility. In such a case, society is inclined to consume more today and to invest less for future consumption potential.

The elasticity of the marginal utility of consumption *θ*, *θ* ≥ 0, determines the gain in utility due to additional consumption, irrespective of the timing of its appearance. It enters the utility function as

11 $$U\left( {c_t} \right) = \left\{ {\begin{array}{*{20}{c}} {\frac{{c_t^{1 - \theta }}}{{1 - \theta }}\,\quad{\rm{for}}\;\theta \,\,\ne\,\, 1} \\ {{\mathrm{ln}}\;c_t\,\quad{\rm{for}}\;\theta = 1}. \end{array}} \right.$$

The calibration of these parameters is controversially discussed in climate economics as they reflect either how decisions shall be formed on account of ethical concerns or how decisions are actually made. Ethical considerations are, for instance, reflected by an almost zero IRSTP, as it assigns future generations’ consumption similar relevance as the current generation’s consumption^24,62^. In contrast, the choice of a higher rate reflects that people usually prefer consuming today rather than postponing it. Likewise, the consumption elasticity parameter can be determined either based on empirical studies^63^ or by answering the normative question of how much importance additional consumption shall have for the society’s well-being^64^.

Together, these two parameters describe the social-welfare-equivalent discount rate *r*, which converts a marginal change in future consumption at time *t* into the welfare-equivalent marginal change in current consumption given by

12 $$\frac{{\partial W_1}}{{\partial C_1}} = \left( {1 + r} \right)^t\frac{{\partial W_1}}{{\partial C_t}}.$$

From this relation, one can derive the Ramsey equation that connects the two parameters with the discount rate *r* as follows

13 $$r \approx \rho + \theta g$$

with the consumption growth rate *g* (cf. Goulder and Williams^64^).

Equations (11) and (12) illustrate that the two parameters influence the weight of the future generations’ well-being for today’s policy. In particular, they influence the importance of protecting against future climate impacts for today’s policy, weighing up the benefits future societies would experience against the emission reduction costs that today’s generation would have to bear. The choice of their values thus is critical to assessments of climate change policy. Furthermore, Eq. (12) shows that they also affect the balance between optimal consumption and thus indirectly optimal investment and can thus change the growth effects that are critical for our results.

The calibration of these parameters is subject to a longstanding debate. According to the descriptive viewpoint taken in DICE^20^, it is critical that the two preference parameters are chosen simultaneously so that the resulting discount rate reflects observed behaviour revealed by market interest rates. In contrast, the prescriptivists^24^ perceive the calibration of the two parameters as an ethical issue. Following now the prescriptive approach, we account for a wide range of possible values. The results of this sensitivity test are shown in Fig. 5 and described in the main text. As explained above, the temperature targets for small discount rates might be estimated to be too high due to the deficient reproduction of the carbon cycle dynamics in DICE. As these targets are well below 2 °C, this implied error does not contradict our general finding that the Paris Agreement could be cost-benefit optimal.

### Reporting summary

Further information on research design is available in the Nature Research Reporting Summary linked to this article.

## Supplementary information

Supplementary Information

Reporting Summary

## Data Availability

The datasets generated and analysed during this study including data shown in the figures are available from the authors upon request.

## Code availability

The code used in this study is available from the authors upon request.
