# Supplementary material for: Paris Climate Agreement passes the cost-benefit test
Source: Nat Commun. 2020 Jan 27;11:110. doi: 10.1038/s41467-019-13961-1 (PMC6985261; doi:10.1038/s41467-019-13961-1)
Supplement: Supplementary file 1 — Supplementary Information [file 41467_2019_13961_MOESM1_ESM.pdf]

Paris Climate Agreement passes the cost-benefit test

Glanemann et al.

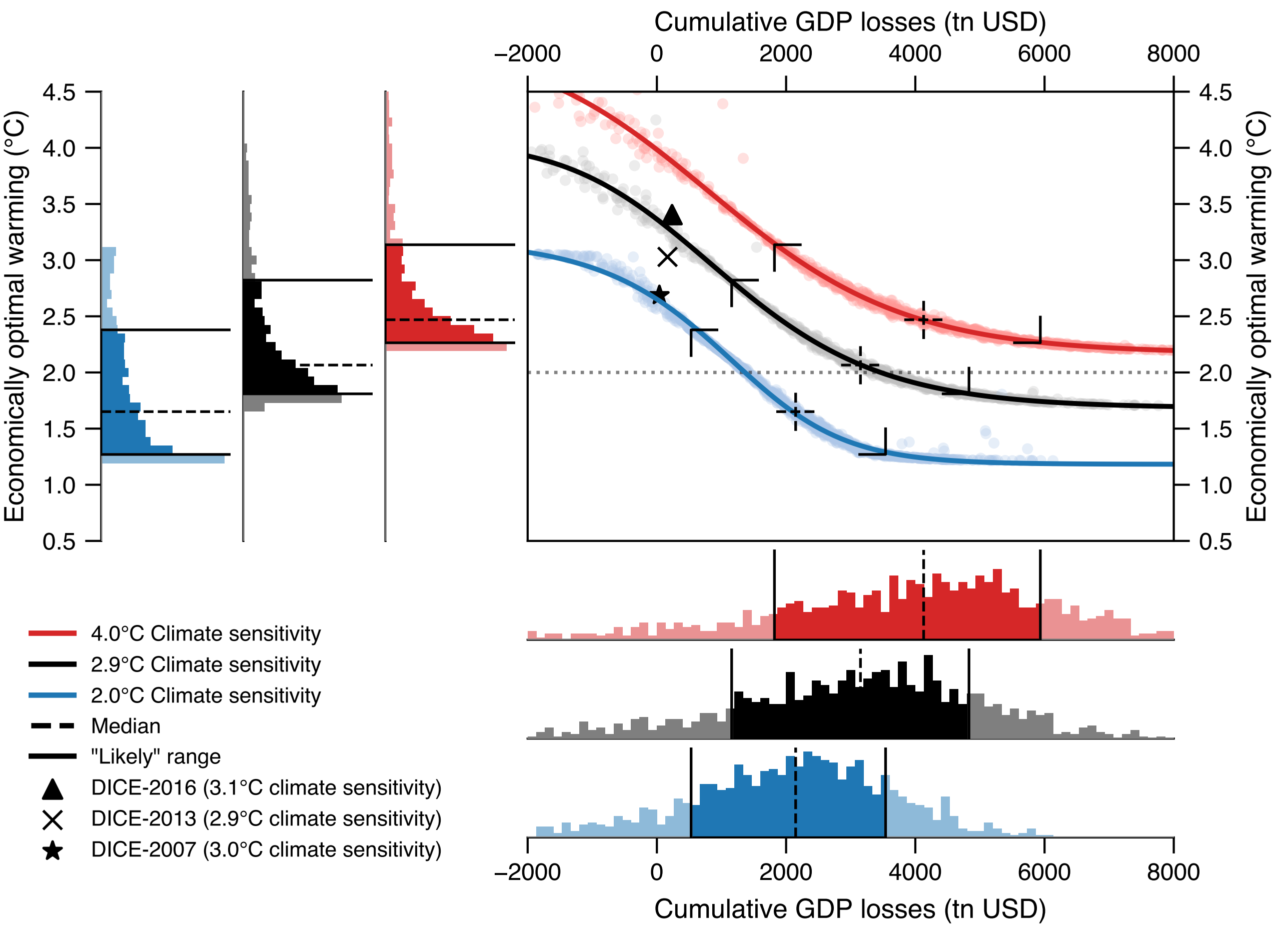

Supplementary Figure 1: Same as Figure 3 but with sampling over years (in the BHM derivation) instead of sampling over countries.

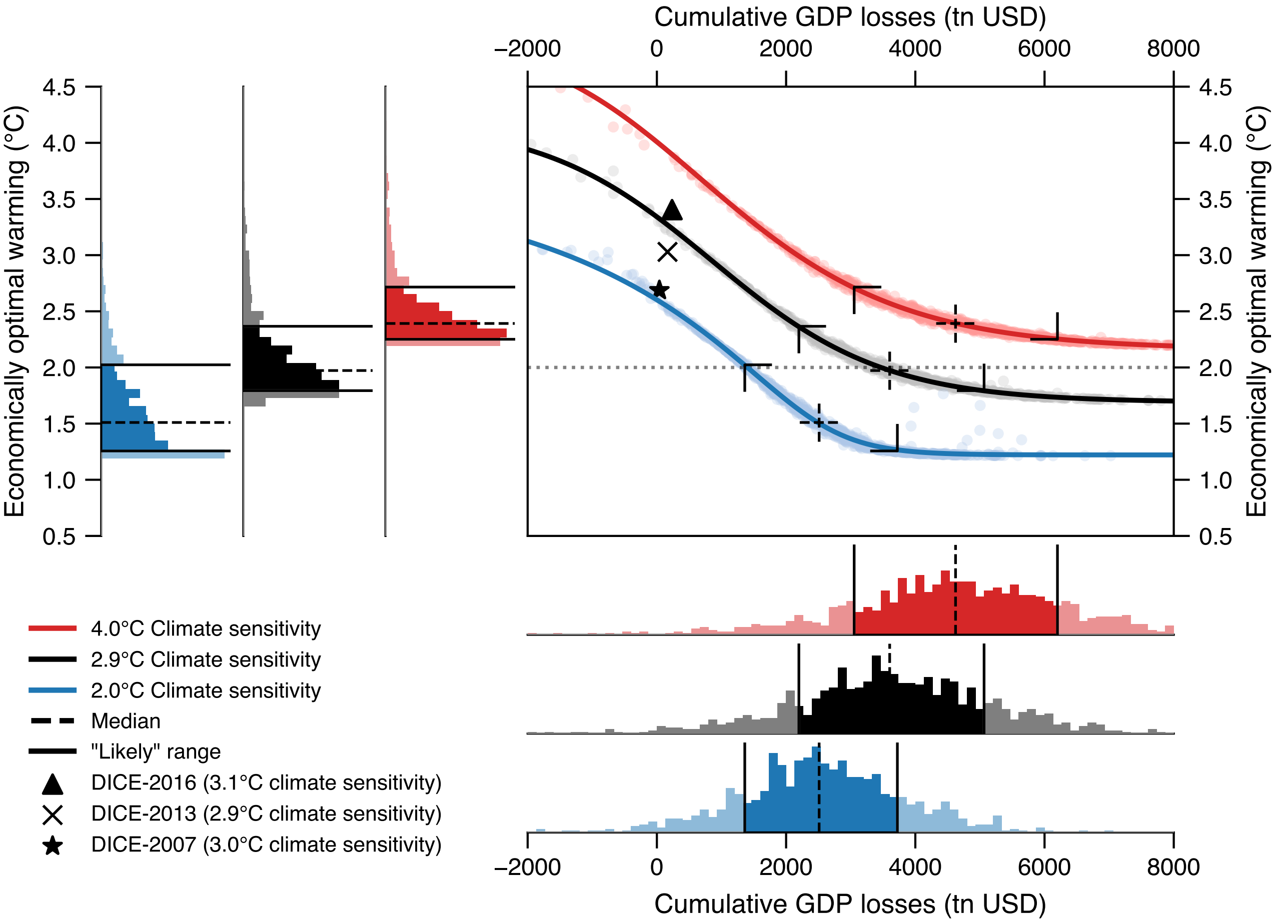

Supplementary Figure 2: Same as Figure 3 but with sampling over 5-years blocks (in the BHM derivation) instead of sampling over countries.

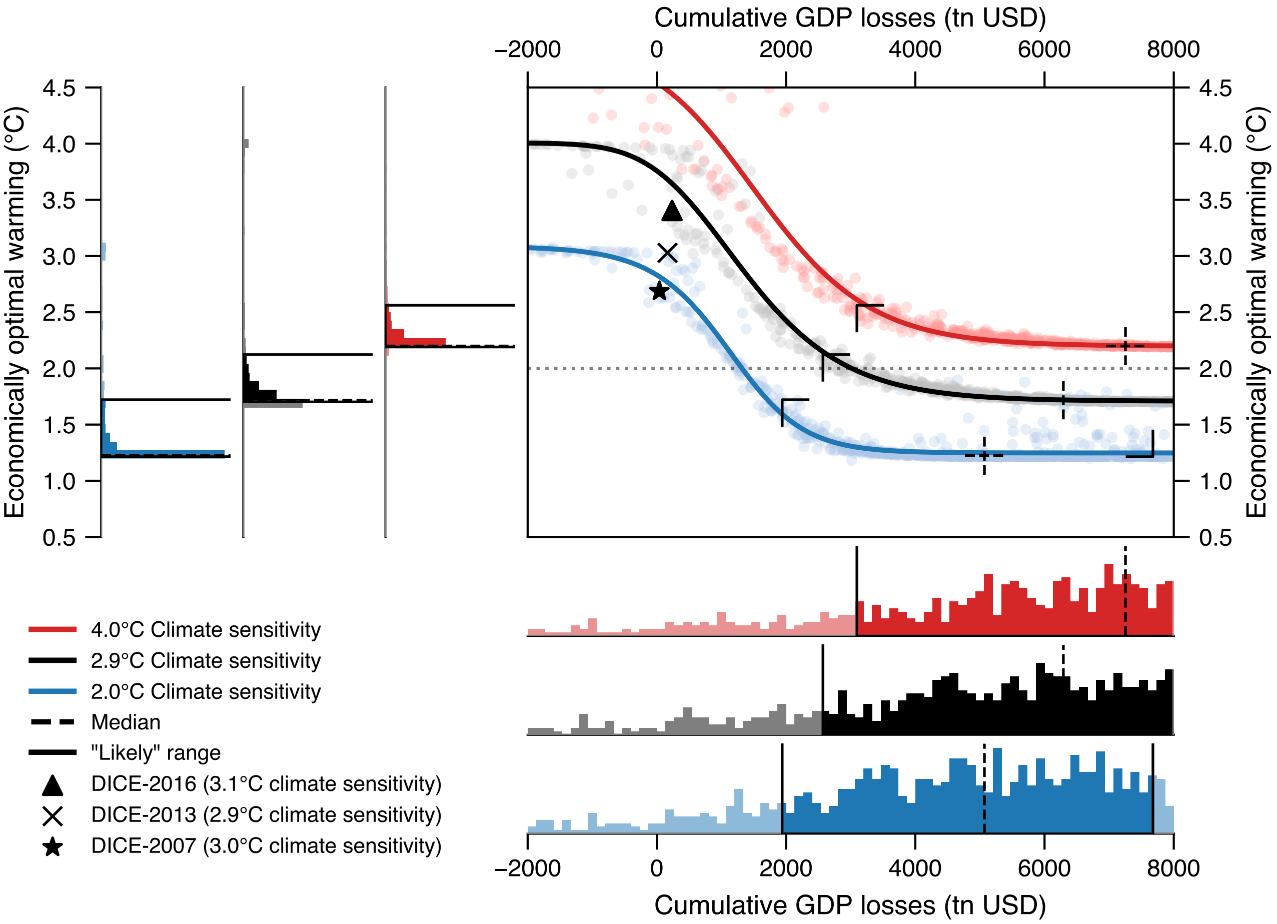

Supplementary Figure 3: Same as Figure 3 but with the assumption that the influence of warming on economic growth is lagged as defined by the *pooled long-run specification* by BHM.

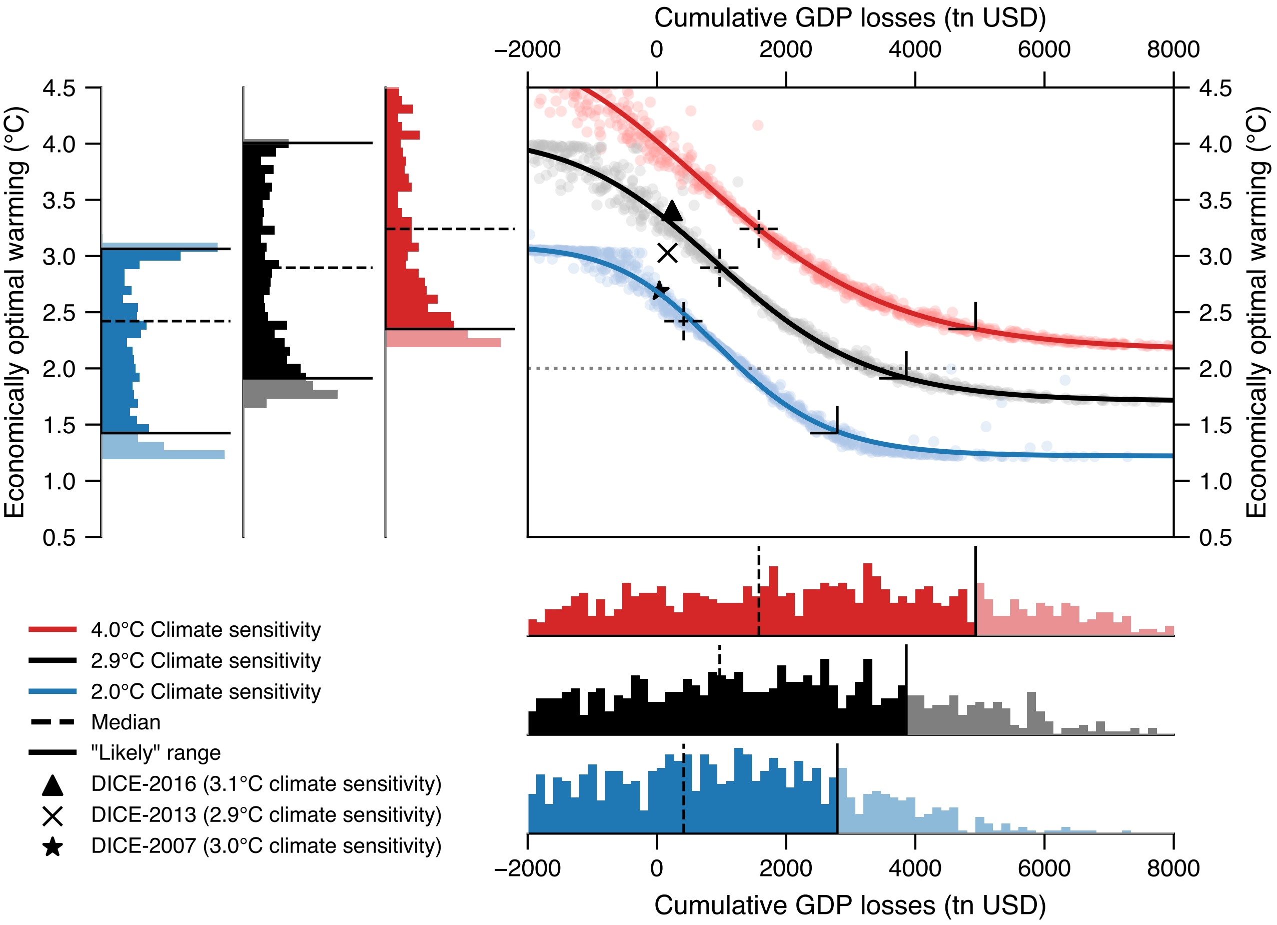

Supplementary Figure 4: Same as Figure 3 but with the assumption that the influence of warming on economic growth is not identical for rich and poor countries as given by the *differentiated short-run specification* by BHM.

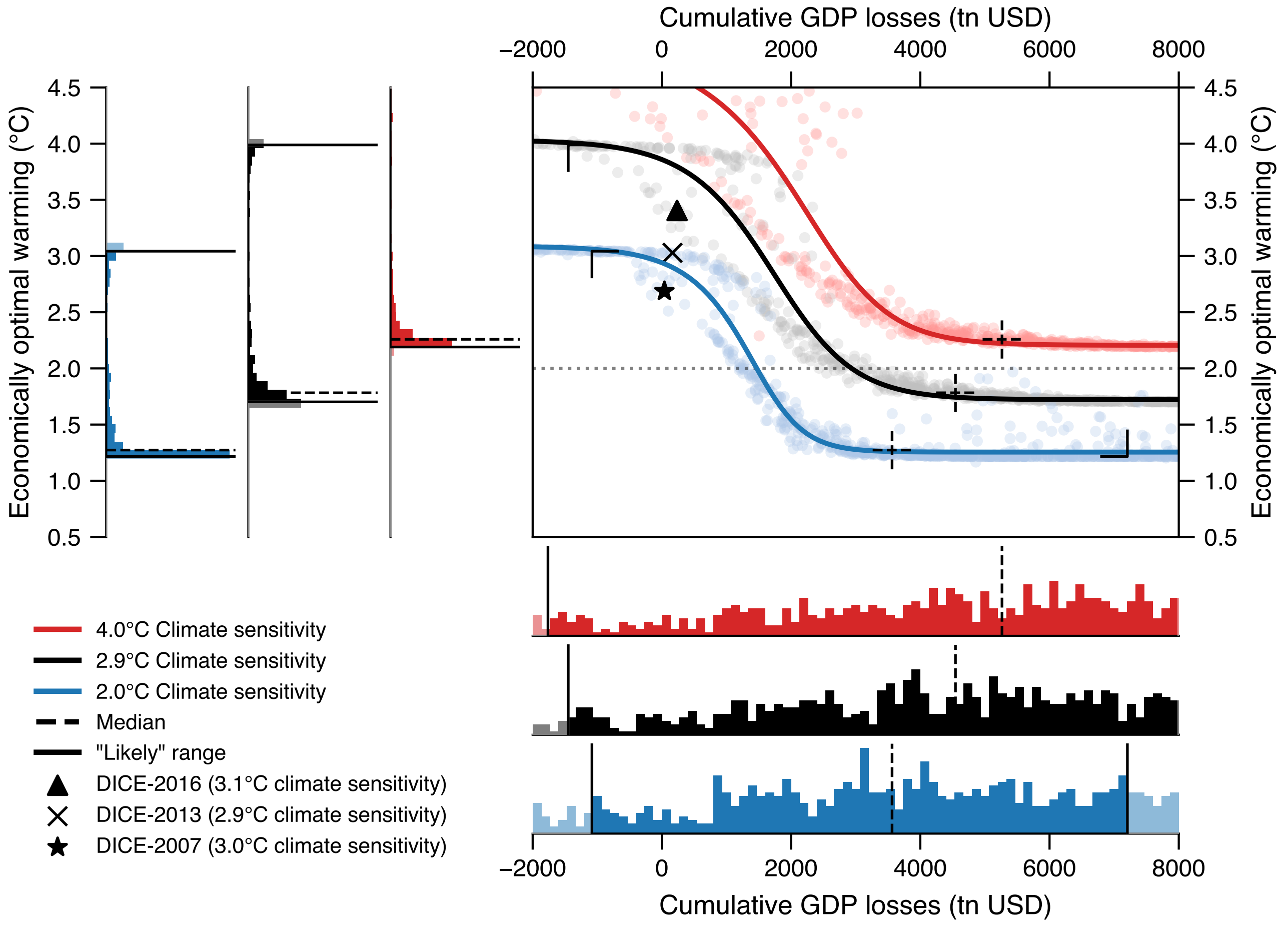

Supplementary Figure 5: Same as Figure 3 but with the assumption that the influence of warming on economic growth is lagged and not identical for rich and poor countries as given by the *differentiated long-run specification* by BHM.

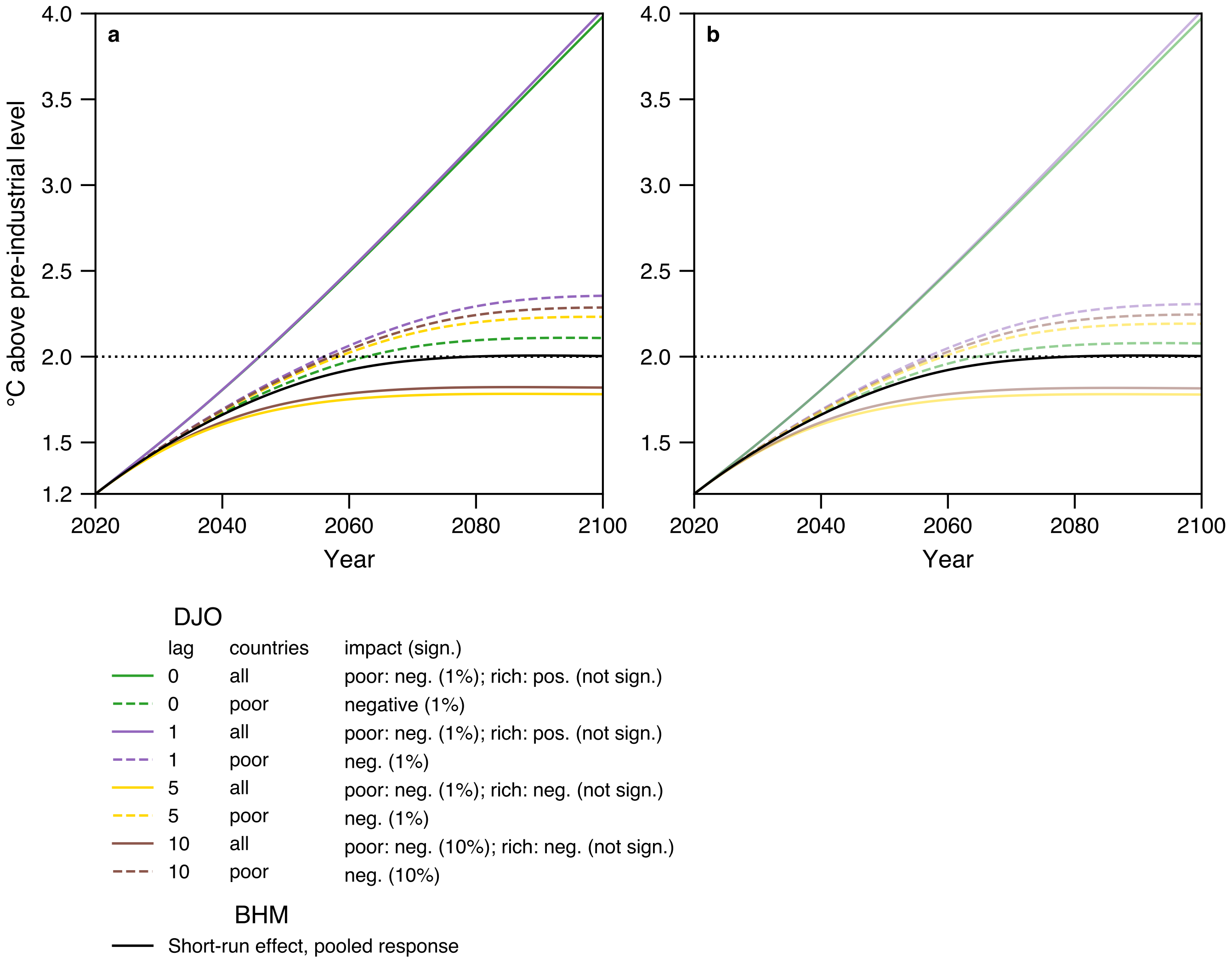

Supplementary Figure 6: The optimal temperature pathways for the DJO estimates resulting from different specifications. We test all lag specifications together with and without the estimation results for rich countries. Given that DICE is a global model, we implement the DJO estimates with the help of assumptions about the poor countries' share in global GDP. **a** the share decreases linearly and **b** the share stays on its rather high level of 1980. The comparison shows that the exact specification of the share does not imply virtually different results.

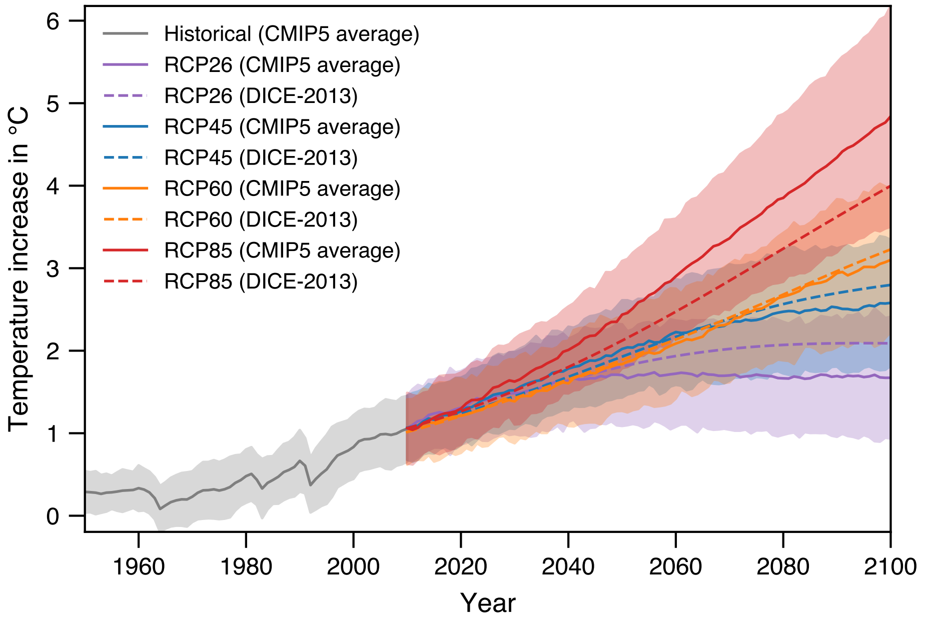

Supplementary Figure 7: Temperature development projected by DICE-2013 (dashed curves) and by the model ensemble used in AR5WG1 (IPCC, *The physical science basis*, 2013) for the four standard Representative Concentration Pathways (RCPs). The change in global annual mean surface air temperature is given relative to pre-industrial times. Solid lines represent the respective average of the CMIP5 ensemble, shadings show the 1.64 standard deviation range around the average (this corresponds to 5% to 95% when assuming a normal distribution of the respective ensemble; cf. Figure AI. 1 in AR5WG1). For the DICE-2013 temperature development RCP-equivalent emission data is used to run the carbon cycle and climate module.

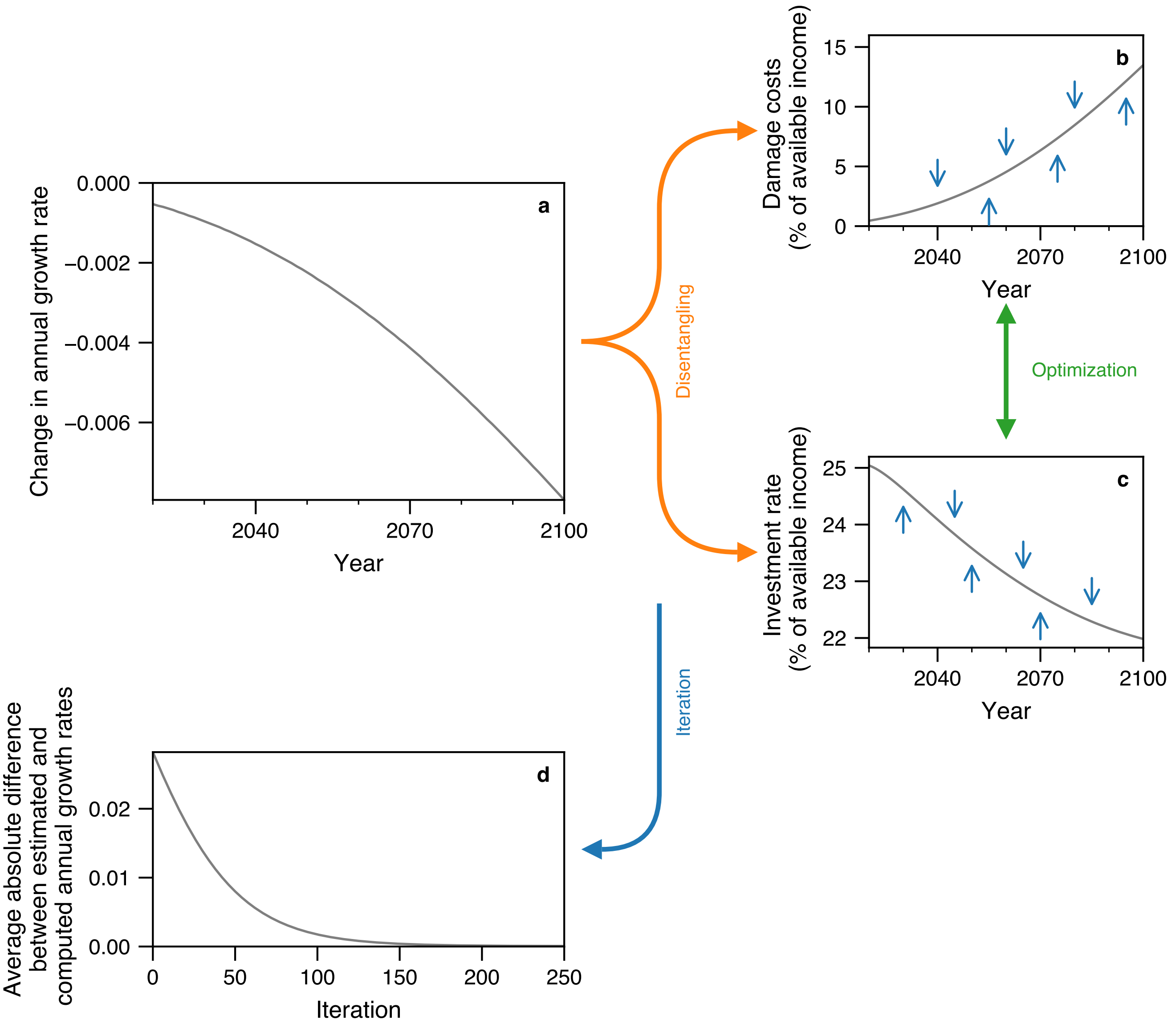

Supplementary Figure 8: Schematic representation of the iterative procedure. a The estimated change in the annual growth rate due to temperature increase is disentangled into **b** the damage function and **c** in its associated optimal investment response in the business-as-usual scenario, which is characterized by inaction of climate policy. **d** The iterated growth rate converges towards the estimated growth rate after approximately 200 iterations.

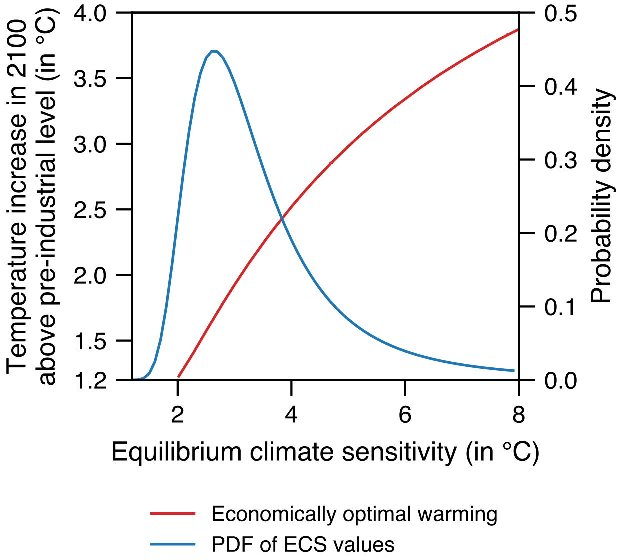

Supplementary Figure 9: Full sensitivity analysis with respect to equilibrium climate sensitivity (ECS) employing the ECS probability distribution (blue curve) of Roe & Baker (*Why Is Climate Sensitivity So Unpredictable?*, Science 80, 629–632, 2007) calibrated to results of a GCM. The red curve gives the economically optimal temperature increase for all ECS values between 2°C and 8°C.

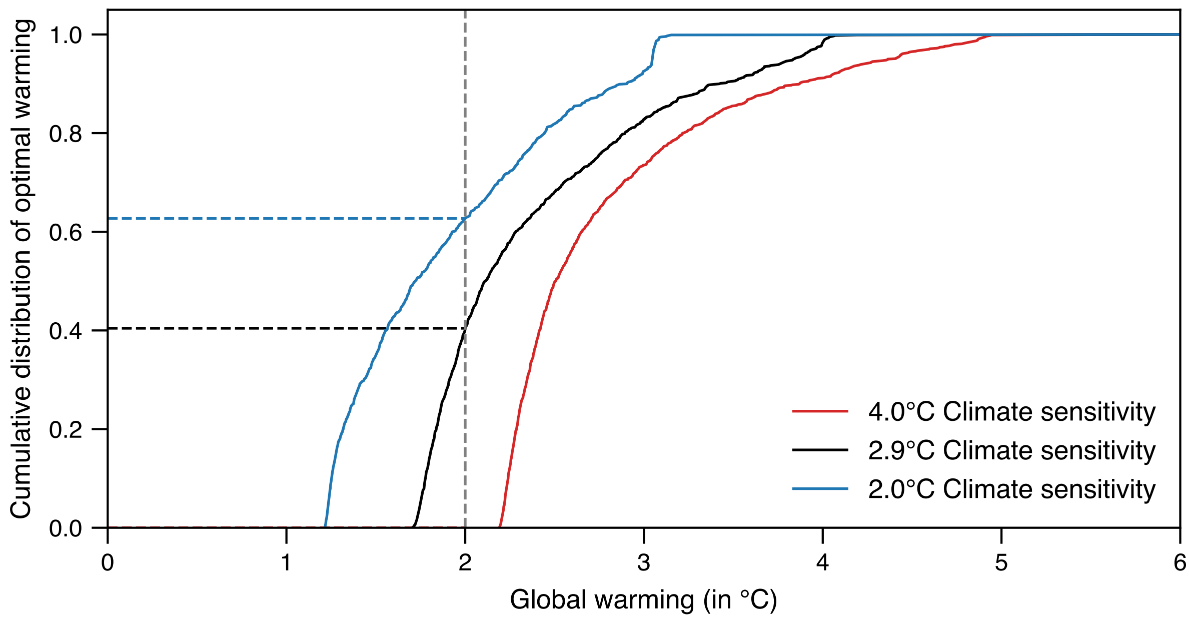

Supplementary Figure 10: Cumulative distribution of the economically optimal end-of-century temperatures given the uncertainty in the economic growth response to temperature estimates. Economic optimality of the temperature target of 2°C or below are implied by 40% (0%, 63%) of the bootstrapped estimates for a 2.9°C (4°C, 2°C) climate sensitivity, respectively.
